# An agonistic anti-Tie2 antibody suppresses the normal-to-tumor vascular transition in the glioblastoma invasion zone

**DOI:** 10.1038/s12276-023-00939-9

**Published:** 2023-02-24

**Authors:** Eunhyeong Lee, Eun-Ah Lee, Eunji Kong, Haemin Chon, Melissa Llaiqui-Condori, Cheon Ho Park, Beom Yong Park, Nu Ri Kang, Jin-San Yoo, Hyun-Soo Lee, Hyung-Seok Kim, Sung-Hong Park, Seung-Won Choi, Dietmar Vestweber, Jeong Ho Lee, Pilhan Kim, Weon Sup Lee, Injune Kim

**Affiliations:** 1 Graduate School of Medical Science and Engineering, Korea Advanced Institute of Science and Technology (KAIST), Daejeon, 34141 Republic of Korea; 2 R&D Center, PharmAbcine Inc., Daejeon, 34047 Republic of Korea; 3 Department of Bio and Brain Engineering, KAIST, Daejeon, 34141 Republic of Korea; 4 Department of Forensic Medicine, Chonnam National University Medical School, Gwangju, 61463 Republic of Korea; 5 Department of Neurosurgery, Samsung Medical Center, Sungkyunkwan University, Seoul, 06351 Republic of Korea; 6 Max Planck Institute for Molecular Biomedicine, D-48149 Muenster, Germany; 7 BioMedical Research Center, KAIST, Daejeon, 34141 Republic of Korea; 8 SoVarGen, Inc., Daejeon, 34051 Republic of Korea; 9 Graduate School of Nanoscience and Technology, Daejeon, 34141 Republic of Korea; 10 KI for Health Science and Technology, KAIST, Daejeon, 34141 Republic of Korea

**Keywords:** Tumour angiogenesis, CNS cancer, Targeted therapies

## Abstract

Tumor progression is intimately associated with the vasculature, as tumor proliferation induces angiogenesis and tumor cells metastasize to distant organs via blood vessels. However, whether tumor invasion is associated with blood vessels remains unknown. As glioblastoma (GBM) is featured by aggressive invasion and vascular abnormalities, we characterized the onset of vascular remodeling in the diffuse tumor infiltrating zone by establishing new spontaneous GBM models with robust invasion capacity. Normal brain vessels underwent a gradual transition to severely impaired tumor vessels at the GBM periphery over several days. Increasing vasodilation from the tumor periphery to the tumor core was also found in human GBM. The levels of vascular endothelial growth factor (VEGF) and VEGF receptor 2 (VEGFR2) showed a spatial correlation with the extent of vascular abnormalities spanning the tumor-invading zone. Blockade of VEGFR2 suppressed vascular remodeling at the tumor periphery, confirming the role of VEGF-VEGFR2 signaling in the invasion-associated vascular transition. As angiopoietin-2 (ANGPT2) was expressed in only a portion of the central tumor vessels, we developed a ligand-independent tunica interna endothelial cell kinase 2 (Tie2)-activating antibody that can result in Tie2 phosphorylation in vivo. This agonistic anti-Tie2 antibody effectively normalized the vasculature in both the tumor periphery and tumor center, similar to the effects of VEGFR2 blockade. Mechanistically, this antibody-based Tie2 activation induced VE-PTP-mediated VEGFR2 dephosphorylation in vivo. Thus, our study reveals that the normal-to-tumor vascular transition is spatiotemporally associated with GBM invasion and may be controlled by Tie2 activation via a novel mechanism of action.

## Brain tumours: antibody blocks blood vessel abnormality

An antibody targeting key signalling pathways could prevent brain tumor vessels from being abnormal, thereby improving drug delivery into tumor tissues. Glioblastoma multiforme is considered one of the most deadly cancers and it invades brain tissue coupled with the loss of vascular integrity. This pathological vascular changes are closely associated with the hyperactivation of a signalling pathway called VEGFR2 and inactivation of another pathway, Tie2. Now, Injune Kim at the Korea Advanced Institute of Science and Technology, Weon Sup Lee at PharmAbcine Inc., both in Daejeon, South Korea, and co-workers have developed a Tie2-activating antibody that effectively stops vascular abnormalization in and around glioblastoma in the spontaneous mouse tumor model. As well as activating Tie2, the antibody suppresses the VEGFR2 pathway. The study therefore reveals a potential strategy for improving glioblastoma treatment through multiple vascular mechanisms.

## Introduction

Cancer progression is intimately associated with blood vessels^1^. Tumor proliferation induces tumor angiogenesis, and blood vessels are the main route through which metastatic tumor cells seed distant organs^2,3^. Although invasion is a hallmark of cancer, whether and how it is associated with blood vessels remains poorly understood. Glioblastoma multiform (GBM) is the most lethal brain tumor because of its invasion of brain tissue, although it rarely exhibits distant metastasis^4^. As excessive vascular abnormalities are a hallmark of GBM^5^, GBM is likely an appropriate tumor type for understanding invasion-associated vascular characteristics.

GBM cells at the tumor periphery infiltrate neighboring tissues diffusely and form an invasion region between the normal brain tissue with the blood‒brain barrier and the main tumor mass with a leaky vasculature^6^. The standard surgical procedure for GBM resection usually prevents the collection of the tumor-infiltrating area for biopsy. In addition, most orthotopic glioma models have been unsuccessful in reproducing the histopathological characteristics and invasive traits of grade IV gliomas. Due to these limitations, the vascular characteristics of and vascular dynamics in the GBM invading zone and the related underlying mechanisms remain largely unknown compared to those of the vasculature in the main tumor mass. Therefore, advanced modeling of GBM invasion is required to understand invasion-related vascular features.

The vascular endothelial growth factor receptor 2 (VEGFR2) and tunica interna endothelial cell kinase 2 (Tie2) signaling pathways are essential for proper brain angiogenesis and blood‒brain barrier genesis during development^7,8^. However, hyperactivation of VEGFR2 and inactivation of Tie2 underlie the vascular pathology in brain tumors^9^. In addition, excessive levels of vascular endothelial growth factor (VEGF) and angiopoietin-2 (ANGPT2), an antagonistic ligand of Tie2, correlate with a poor prognosis in patients with GBM^10–12^. Therefore, these ligands have been considered therapeutic targets in GBM. Although the FDA finally approved bevacizumab, an anti-VEGF neutralizing antibody, for the treatment of patients with recurrent GBM^13^, its therapeutic benefit is only transient, and overall survival is not improved^14^. Several ANGPT2 inhibitory agents for Tie2 activation have also produced unsatisfactory outcomes in clinical trials for the treatment of GBM^15,16^, demanding alternative methods of Tie2 activation.

In the present study, we established a mouse model of spontaneous GBM with invasive traits and tried to elucidate the poorly understood invasion-associated vascular characteristics. We further explored whether and how the VEGFR2 and Tie2 signaling pathways are involved in the vascular pathology associated with GBM invasion.

## Materials and Methods

### Mice

Mice with the *LSL-EGFR*_*VIII*_ (NCI), *LSL-EGFRwt* (NCI), *LSL-tdTomato* (Jackson Laboratory) and *LSL-Luciferase* (Jackson Laboratory, Bar Harbor) alleles were maintained on a C57BL/6 background. All mouse lines were backcrossed onto the C57BL/6 background for at least 10 generations. Mice were anesthetized with 80 mg/kg ketamine and 12 mg/kg xylazine, and their brains were harvested for further analyses. Every mouse was reared in a specific pathogen-free animal facility at the Korea Advanced Institute of Science and Technology (KAIST). All animal experiments were approved by the Animal Care Committee of the Korea Advanced Institute of Science and Technology (KA2018-41). All procedures and animal handling were performed following the ethical guidelines for animal studies.

### Spontaneous GBM mouse model and treatment regimens

A hybrid plasmid containing CRISPR/Cas9 and Cre-expressing constructs was used to inactivate the target genes *TP53* and *PTEN* and overexpress *EGFR*_*VIII*_, as described earlier^17^. Postnatal day 0–2 pups were placed onto a glass Petri dish prechilled to 4 °C. After anesthetization, the pups were subjected to intraventricular injection. The ideal site of injection was approximately equidistant from the lambdoid suture and the right eye and 2 mm lateral to the sagittal suture. Approximately 2 μl of plasmid solution (> 2.5 μg/μl) containing 1% Fast Green (F7252, Sigma-Aldrich, St. Louis) was injected into the right lateral ventricle using an aspirator tube for calibrated microcapillary pipettes (A5177, Sigma-Aldrich). If the plasmid solution was properly inserted into the lateral ventricle, the green color of Fast Green was observable in the shape of the lateral ventricle. Only appropriately injected mice were eligible for the next electroporation step. The electroporation conditions were set as follows: 5 square pulses of 50 msec/pulse at 100 volts, with 950 msec intervals (ECM830, BTX Harvard Apparatus, Holliston) and 3 mm tweezer electrodes (CUY650P3, Nepa Gene, Chiba). The positive electrode was placed on the dorsal lateral wall of the right lateral ventricle of the pup near the ear ipsilateral to the site of plasmid injection, and the negative electrode was positioned at the contralateral hemisphere ventral lateral to the pup’s snout. After electroporation, somatic mutation occurred within 48–72 h and induced spontaneous glioblastoma exclusively in EGFR mutant mice from 5 weeks to 20 weeks. Cells with mutations introduced by the CRISPR/Cas9 and Cre/loxP systems were identified by using the td-tomato reporter gene. To block VEGFR2 and PD-1 and activate Tie2, 40 mg/kg DC101, 8 mg/kg anti-PD-1 antibody, 40 mg/kg 4E2 (PharmAbcine Inc., Daejeon), and equal amounts of the corresponding control antibodies were intraperitoneally injected into tumor-bearing mice twice a week. To inhibit VE-PTP, 40 mg/kg AKB-9778 (razuprotafib; HY-109041, MedChemExpress, Monmouth Junction) was subcutaneously injected into GBM-bearing mice twice daily for 5 days as described previously^18^.

### Cells

Human umbilical vein ECs (HUVECs, ATCC, Manassas) were seeded in confluent conditions (8 × 10^5^ cells/well) in 6-well plates. HUVECs cultured in Human Endothelial Medium (LL-0003, Lifeline Cell Technology, Frederick) for 24 h were pretreated with 100 ng/ml human angiopoietin-1 (ANGPT1, 923-AN/CF, R&D Systems, Minneapolis), 100 ng/ml angiopoietin-2 (ANGPT2, 623-AN/CF, R&D Systems), 30 μg/mL 4E2, or 2.5 μM AKB-9778 for 20 min and were then treated with 100 ng/mL human VEGF-165 protein (293-VE/CF, R&D Systems) for 10 min. When indicated, CHO-K1 cells expressing human Tie2 or mouse Tie2 were maintained in DMEM (Thermo Fisher, Waltham) supplemented with 10% FBS and 200 ng/ml hygromycin (Thermo Fisher).

### Competitive binding of 4E2 and angiopoietins

An Octet Red 96e system (Sartorius, Goettingen) equipped with Ni-NTA sensors was used to measure the competitive binding of 4E2 and angiopoietins. The human anti-Tie2 antibody (50 nM)-immobilized biosensor was immersed in a well containing 100 nM ANGPT1 or ANGPT2 in phosphate-buffered saline (PBS) by centrifugation at 1,000 rpm for the first binding step. After the sensorgram plateaued (up to 2200 sec), the ANGPT1-bound sensor was moved to a well containing PBS buffer without a reagent, 100 nM ANGPT1 (or ANGPT2) or 27.2 nM 4E2 (the second binding step). To evaluate the competition between ANGPTs and 4E2, PBS without ANGPTs was also tested in the first binding step. Additionally, the first and second binding steps were reversed to evaluate the binding characteristics of 4E2.

### Immunocytochemistry

To assess Tie2 localization and FoxO1 translocation, HUVECs were seeded in 4-well slide chambers (Lab-TekII, 154526, Thermo Fisher) and maintained in Vasculife medium for 24 h. When fully confluent, the HUVECs were treated with 10 μg/ml 4E2, 100 ng/ml ANGPT1, or 100 ng/ml VEGF for 24 h. Thereafter, the cells were fixed with 4% paraformaldehyde (PFA) for 10 min, permeabilized with 0.1% Triton X-100 in PBS, blocked with 1% BSA in PBS for 40 min, and incubated at 4 °C overnight with primary antibodies: Alexa Fluor 488-conjugated mouse anti-VE-cadherin (16B1, 53-1449-42, Thermo Fisher) or rabbit anti-FoxO1 (C29H4, 2880, Cell Signaling Technology, Danvers). The cells were then incubated with an Alexa Fluor 488-conjugated goat anti-rabbit (A11008, Thermo Fisher) secondary antibody for 1 h, stained with DAPI (564907, BD Biosciences, Franklin Lakes) for 5 min, and embedded in mounting medium (F4680, Sigma-Aldrich). Images were acquired by fluorescence microscopy (EVOS-M5000, Thermo Fisher).

### Immunoprecipitation

HUVECs were pretreated with 30 μg/ml 4E2 for 20 min and then treated with VEGF for 10 min. Cells were harvested by scraping with cold PBS and lysed in 1% NP-40 lysis buffer (20 mM HEPES (pH 7.4), 150 mM NaCl, 1 mM EDTA, and 1% NP-40) supplemented with PhosStop (4906845001, Roche, Basel) and cOmplete (11836170001, Roche) phosphatase and protease inhibitors for 10 min. The lysate was incubated overnight with an anti-human VE-PTP antibody^19^, and then Protein-A/G agarose beads (sc-2003, Santa Cruz Biotechnology, Dallas) were added and incubated with inversion at 4 °C for 3 h. The beads were washed three times with 1% NP-40 lysis buffer. Proteins were transferred to a PVDF membrane and analyzed by immunoblotting using the same antibodies described for Western blot analysis.

### Histological analysis

After anesthesia, transcardial perfusion was performed on the mice with PBS followed by 4% paraformaldehyde (PFA, Merck, Rachway). For H&E staining, human patient GBM tissues, mouse brain tumors and normal mouse brains were fixed overnight at 4 °C with 4% PFA, embedded in paraffin, and cut into 3-μm sections. Images of H&E staining were acquired using an Axio Scan.Z1 slide scanner (Carl Zeiss, Oberkochen). For immunofluorescence staining, samples were fixed with 4% PFA and dehydrated in PBS at 4 °C overnight. Samples were sectioned at a 60 μm thickness using a vibrating microtome (Leica Biosystems, Wetzlar). Tissue sections were blocked in PBST (0.3% Triton X-100 in PBS) with 5% goat or donkey serum. The following primary antibodies were used for immunostaining of mouse samples: anti-CD31 (hamster monoclonal, clone 2H8), anti-PDGFRβ (rat monoclonal, clone APB5), anti-collagen type IV (rabbit polyclonal, ab6586, Abcam, Cambridge), anti-VEGFR2 (goat polyclonal, AF644, R&D Systems), anti-VEGF_164_ (goat polyclonal, AF-493-NA, R&D Systems), anti-Tie2 (goat polyclonal, AF762, R&D Systems), anti-ANGPT2 (rabbit polyclonal, ab8452, Abcam), anti-VE-cadherin (goat polyclonal, AF1002, R&D Systems), anti-Caveolin1 (rabbit polyclonal, sc894, Santa Cruz Biotechnology), anti-PLVAP (rat monoclonal, 550563, BD Biosciences), anti-pTie2 (Tyr992, rabbit polyoclonal, AF2720, R&D Systems), anti-pVEGFR2 (Tyr1175, rabbit monoclonal, 2478, Cell Signaling), anti-pAkt (Ser473, rabbit monoclonal, 4060, Cell Signaling), anti-ESM1 (goat polyclonal, AF1999, R&D Systems), anti-Claudin5 (rabbit polyclonal, 34-1600, Invitrogen, Waltham), anti-CD45 (rat monoclonal, 550539, BD Biosciences), anti-F4/80 (rat monoclonal, 14-4801-82, eBioscience, San Diego), anti-CD4 (rat monoclonal, 550280, BD Biosciences), anti-CD8α (rat monoclonal, 558733, BD Biosciences), anti-VE-PTP (rat monoclonal, clone 109.1)^20^, and anti-HIF1-α (rabbit polyclonal, NB100-134, Novus Biologicals, Englewood). The following primary antibody was used for immunostaining of human samples: anti-CD31 (rabbit polyclonal, ab28364, Abcam). After several washes with PBST, sections were incubated with Alexa Fluor 488-, 594-, or 647-conjugated secondary antibodies (Jackson ImmunoResearch, West Grove). All antibodies used were validated for the species and application. Nuclei were stained with DAPI (Invitrogen). The samples were then mounted with fluorescent mounting medium (Vector, Olean), and immunofluorescence images were acquired using LSM780 and LSM800 confocal microscopes (Carl Zeiss).

### Assessment of vascular leakage and perfusion

To assess vascular leakage, Evans blue solution (2% v/w, 100 µl, Sigma-Aldrich) was injected intravenously 6 h before brains were harvested. To assess vascular perfusion, DyLight 649–conjugated tomato lectin (0.1 mg/200 μl, DL-1178, Vector) was injected intravenously 10 min before brains were harvested. Mice were anesthetized and perfused transcardially with PBS followed by 4% PFA to remove circulating Evans blue and lectin.

### Statistical analysis

Statistical analysis was performed using GraphPad Prism 8.0 (GraphPad Software). Values are presented as the means ± standard deviations (SDs) or standard errors of the mean (SEMs) unless otherwise indicated. For continuous data, statistical significance was determined with Student’s t test or the Mann‒Whitney U test. Changes in body weight and tumor volume according to the date were analyzed using the Bonferroni multiple comparison test after two-way ANOVA, and the tumor weight on the final day was analyzed using Dunnett’s multiple comparison test after one-way ANOVA. A *P* value of less than 0.05 was considered statistically significant.

## Results

### Somatic mutations in the mouse brain lead to spontaneous GBM

**Fig. 1:**
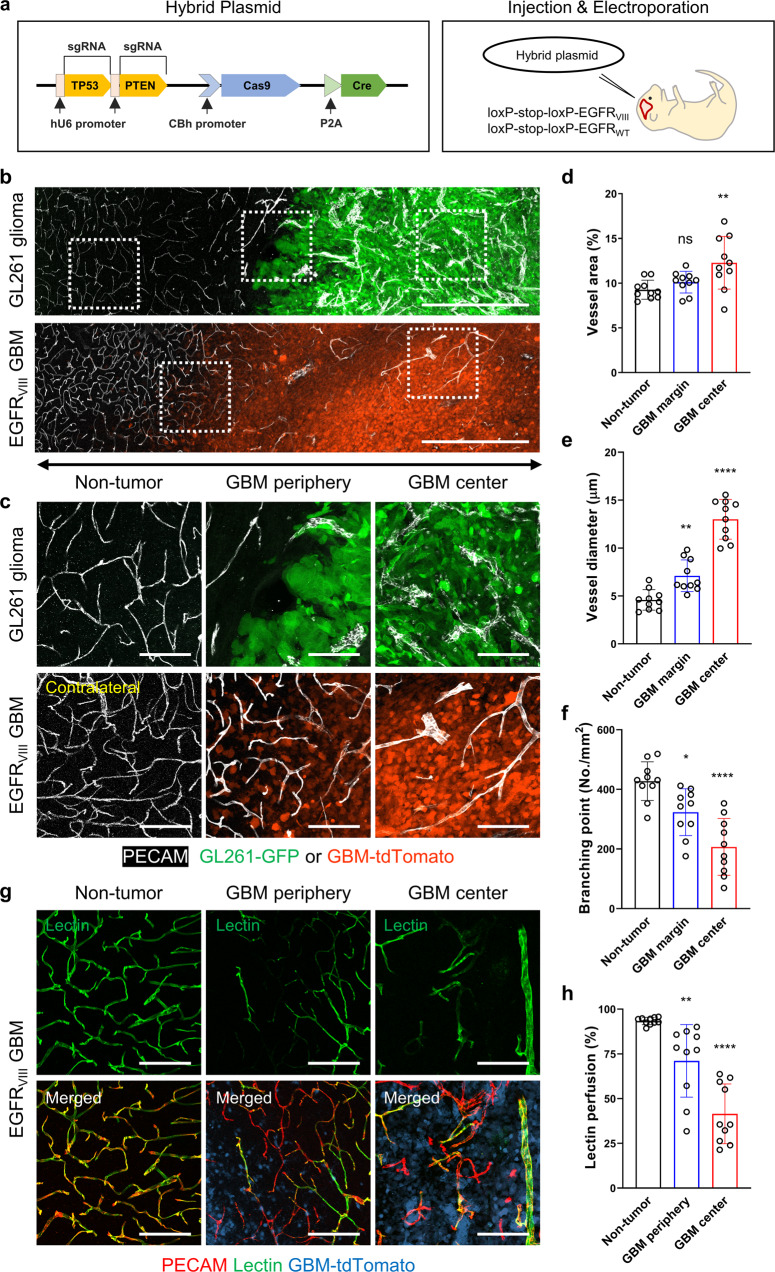
Gradual normal-to-tumor vascular transition through the GBM invasion zone. **a** Hybrid plasmid for both CRISPR/Cas9-mediated gene inactivation and ectopic *Cre* expression (left). This plasmid was delivered into the brain cells of neonatal mice harboring the *EGFR*_*VIII*_ gene cassette (right). **b** Panoramic images of vascular changes along the radial axis in GL261 glioma (upper) and EGFR_VIII_ GBM (lower). **c** Magnified images of the boxed area in (**b**). **d**–**f** Quantification of vascular morphology: (**d**) vessel area, (**e**) vessel diameter, and (**f**) vessel branching points. Note the increase in abnormalities toward the tumor center. **g** Images of lectin perfusion and **h** quantification of lectin-perfused vessels. Scale bars: 500 µm **b**, 100 µm **c**, **g**. ns not significant; *, *P* < 0.05; **, *P* < 0.01; ****, *P* < 0.0001 vs. nontumor tissues.

Although the vascular abnormalities characterizing GBM have historically been an interesting research topic, most previous studies used orthotopic glioma models, which only weakly reproduce the histopathological characteristics of grade IV glioma^21^. To study the vasculature in models reproducing human GBM, we tried to generate a mouse model of spontaneous GBM by using a hybrid plasmid for both CRISPR/Cas9-mediated gene inactivation and Cre recombinase-mediated gene induction. In brief, the tumor suppressor genes *TP53* and *PTEN* were inactivated, and *EGFR*_*VIII*_, which carries a somatic mutation common in human GBM, was overexpressed (Fig. 1a). The use of Cre recombinase also allowed the monitoring of GBM cells ex vivo and in vivo by triggering the expression of reporter genes in transgenic mouse models harboring loxP-STOP-loxP-*tdtomato* and loxP-STOP-loxP-*luciferase* cassettes (Supplementary Fig. 1a). This hybrid plasmid for inducing genetic changes in somatic cells was electroporated into the lateral ventricle of neonatal *EGFR*_*VIII*_ (*EGFR*^*VIII*/*VIII*^) or *EGFR*_*WT*_ homozygous (*EGFR*^*WT*/*WT*^) mice. All *EGFR*^*VIII*/*VIII*^ mice developed brain tumors and cerebral hemorrhage, with a median survival time of 10.5 weeks after electroporation, whereas none of the *EGFR*^*WT*/*WT*^ mice died during the experiment or showed any sign of brain tumors (Supplementary Fig. 1b, c).

Histopathological analysis showed that the brain tumors that developed in *EGFR*^*VIII*/*VIII*^ mice exhibited features of human grade IV GBM, such as necrosis with or without pseudopalisading, microvascular proliferation, a glomeruloid vascular structure, and diffuse tumor invasion (Supplementary Fig. 1e–h). However, the orthotopic GL261 glioma model, which has been considered a model of high-grade glioma, presented no signatures of such grade IV traits, although it exhibited malignancy, as shown by the nuclear atypia and mitotic activity (Supplementary Fig. 1i, j). On T2-weighted magnetic resonance imaging, brain tumors in *EGFR*^*VIII*/*VIII*^ mice had a heterogeneous pattern of increased signal intensity indicative of necrosis and edema, whereas GL261-derived gliomas exhibited a uniform signal intensity (Supplementary Fig. 1d). Based on the histopathological and radiological characteristics, we hereafter refer to the spontaneous brain tumors that developed in *EGFR*^*VIII*/*VIII*^ mice as EGFR_VIII_ GBM.

### Brain vessels undergo a normal-to-tumor transition in the GBM invasion zone

To characterize GBM vessels, we compared the vascular morphology of EGFR_VIII_ GBM, GL261 glioma, and nontumor brain tissues. EGFR_VIII_ GBMs and GL261-derived gliomas had common vascular features distinct from those of nontumor brain vessels, such as an enlarged diameter and a decreased number of branch points (Supplementary Fig. 2a, d–f). Although both tumors had disorganized vascular networks with irregular spacing and an abnormal morphology (Supplementary Fig. 2a), EGFR_VIII_ GBMs exhibited not only greater vascular heterogeneity, as seen by their wider ranges of vascular measures, but also a gradual normal-to-tumor vascular transition (Supplementary Fig. 2d–f). Both tumor types exhibited minimal vascular sprouting and very few ESM1-positive tip cells, cardinal signatures of tumor angiogenesis, at the tumor periphery (Supplementary Fig. 2i). The number of PDGFRβ-positive pericytes and amount of collagen type IV (ColIV)-positive basement membrane (BM) were greatly decreased in GL261 glioma vessels but appeared to be maintained in EGFR_VIII_ GBM vessels at levels equivalent to those in nontumor vessels (Supplementary Fig. 2b, c). In contrast to the lack of extravasation of intravenously infused Evans blue (EB) in normal brain tissue, EB leakage was abundant in tissues of both tumor types, indicating impaired vascular function in tumors (Supplementary Fig. 2g, h). These results indicate that EGFR_VIII_ GBM is characterized by heterogeneously disorganized vascular remodeling and enlarged vessels rather than by aggressive tumor angiogenesis and periendothelial denudation.

Orthotopic GL261 gliomas had demarcated borders, and tumor vessels were distinguished from brain vessels in peripheral nontumor tissue (Fig. 1b, c). In contrast, spontaneous EGFR_VIII_ GBMs had indistinct boundaries with diffuse tumor infiltration into the surrounding tissue (Fig. 1b, c). Blood vessels in the GBM infiltration region proximal to the main tumor mass presented noticeable dilation and reduced branching, but not vessel area, compared to distant nontumor brain vessels (Fig. 1d–f). Consistent with this observation, comparisons of paired samples confirmed the greater enlargement of vessels in the center than in the periphery in GBM patients (Supplementary Fig. 3a–d). Peripheral vessels in the tumor invasion region also exhibited poor lectin perfusion in contrast to the high level of lectin binding to nontumor vessels (Fig. 1g, h). In addition, extravasated EB was substantially detected in the peripheral region of GBMs, but there was no detectable leakage in the peripheral region of GL261 gliomas (Supplementary Fig. 2h). Importantly, the degree of vascular abnormalities in the tumor center was increased compared to that in the tumor invasion zone (Fig. 1e, f, h). Thus, the vasculature in the GBM invasion zone was characterized by a transition from normal brain vessels to severely impaired tumor vessels.

### The levels of VEGF and VEGFR2 but not ANGPT2 correlate with the extent of vascular abnormalities

**Fig. 2:**
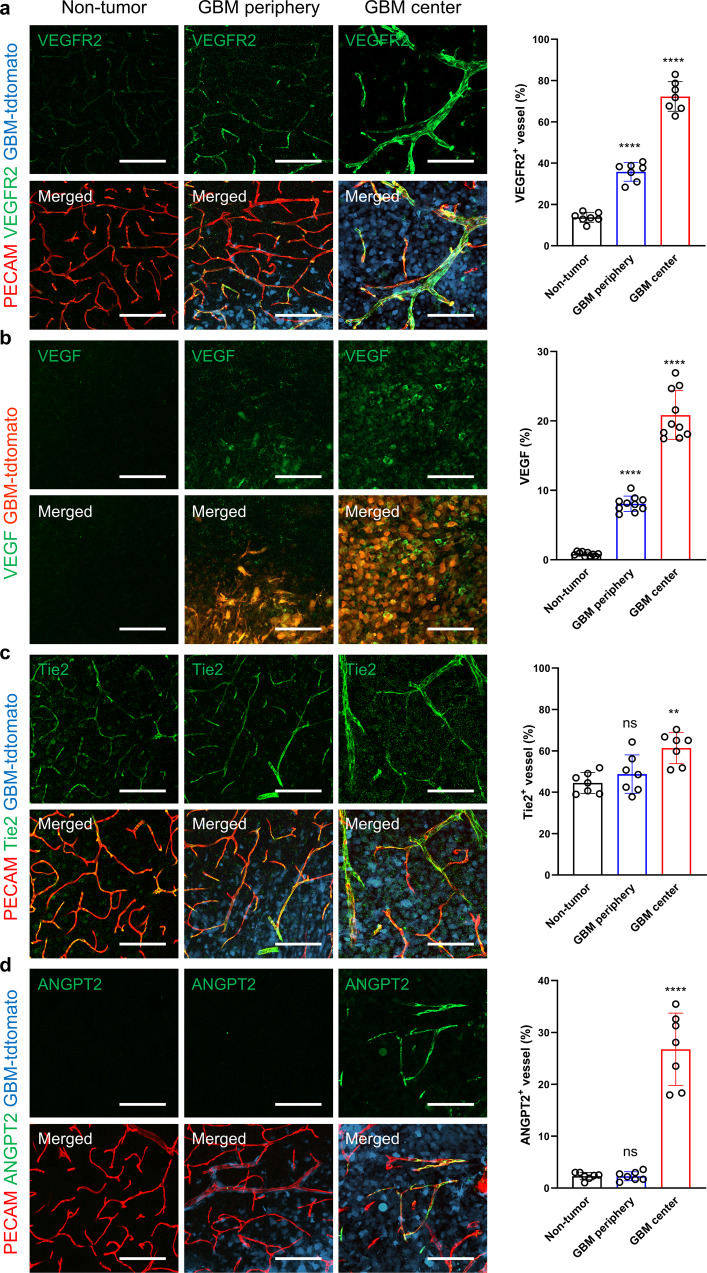
Levels of VEGF and VEGFR2 correlate spatially with the normal-to-tumor vascular transition. **a**–**d** Immunostaining images of nontumor brain tissue and the periphery and center of EGFR_VIII_ GBM tumors and quantification of staining: (**a**) VEGFR2, (**b**) VEGF, (**c**) Tie2, and (**d**) ANGPT2. Scale bars: 100 µm (**a**–**d**). ns not significant; **, *P* < 0.01; ****, *P* < 0.0001 vs. nontumor tissues.

Dysregulation of VEGF/VEGFR2 and ANGPT2/Tie2 signaling leads to vascular abnormalities in various tumor models^22,23^. To understand whether these pathways are implicated in GBM-related vascular changes, we immunostained brain tissues containing EGFR_VIII_ GBM for VEGFR2, VEGF, Tie2, and ANGPT2. VEGFR2 immunoreactivity was relatively modest in <10% of the nontumor brain vessels, whereas the levels and proportions of immunostaining were increased in vessels at the tumor periphery and were far greater in the tumor center (Fig. 2a and Supplementary Fig. 2j). Notably, VEGFR2 was expressed in some phenotypically normal vessels at the GBM periphery. VEGF expression colocalized mostly with tdTomato-positive GBM cells, although some VEGF expression was detected in nontumor cells. The VEGF level was approximately 6-fold greater in the tumor periphery than in nontumor tissues and was further increased in the tumor center (Fig. 2b). These results suggest that VEGF secreted from GBM cells can lead to vascular changes by activating VEGFR2 signaling in nearby vessels. In contrast, both Tie2 and ANGPT2 were specifically expressed in vessels. There was no discernable difference in the endothelial Tie2 level between nontumor tissues and the tumor periphery, but it was increased slightly in the tumor center (Fig. 2c). ANGPT2 was rarely expressed in nontumor brain tissues and peripheral GBM regions but was detected in approximately 25% of tumor vessels in the tumor center (Fig. 2d). These findings indicate a spatial correlation between the levels of VEGF and VEGFR2 and the extent of vascular remodeling in EGFR_VIII_ GBM, suggesting that ANGPT2/Tie2 signaling may be associated with vascular changes in the GBM center.

### GBM peripheral vessels undergo VEGFR2-dependent dynamic remodeling

**Fig. 3:**
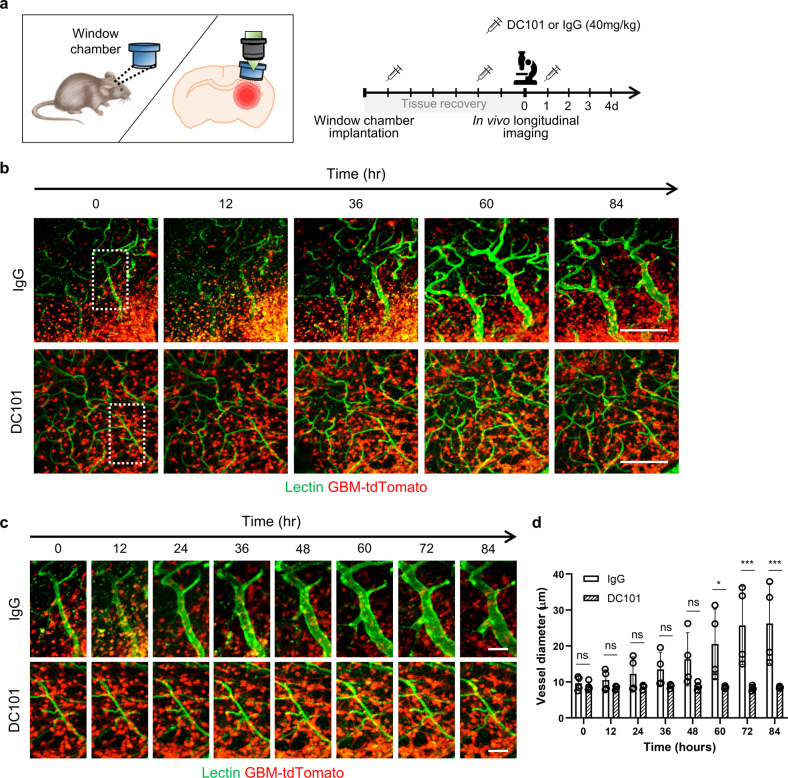
Progressive vasodilation associated with GBM invasion depends on VEGFR2 signaling. **a** Schematic showing the cranial window chamber and experimental schedule with antibody treatment for intravital imaging. **b**–**d** Longitudinal vascular images in EGFR_VIII_ GBM after control IgG or DC101 treatment. Vessels were stained for perfused lectin. **c** Magnified images of the boxed area in (**b**) at a shorter time interval. **d** Quantification of vessel diameter in IgG-treated (*n* = 4) and DC101-treated (*n* = 4) mice. Scale bars: 200 µm (**b**), 50 µm (**c**). ns not significant; *, *P* < 0.05; ***, *P* < 0.001.

Brain vessels dilate or contract rapidly and sensitively in response to metabolic changes in nearby neuronal cells, leading to the concept of neurovascular coupling^24^. As enlarged vessels were found in the GBM invasion zone, we were interested in the speed at which brain vessels dilate upon tumor invasion. To monitor the vascular dynamics at the GBM periphery in deep brain regions below the subcortex, we surgically removed the cortical regions and implanted a tube imaging window chamber over the GBM region (Fig. 3a and Supplementary Fig. 4a–c). For intravital imaging of blood vessels, fluorescently labeled lectin, which can bind to the luminal surface of endothelial cells (ECs), was intravenously infused into mice bearing tdTomato–expressing EGFR_VIII_ GBM tumors. Time-lapse imaging captured the dynamic process of vasodilation in the GBM infiltration zone (Fig. 3b-d). Although vasodilation was a major event (Supplementary Fig. 4d), some vessels underwent constriction, consistent with the decreased vessel branching (Supplementary Fig. 4e–g). These findings indicate that the major initial vascular change is temporally progressive remodeling based on vasodilation and vasoconstriction in the GBM invasion zone.

As VEGFR2 and VEGF were detected at the GBM periphery, we investigated whether the VEGF pathway is responsible for vascular remodeling in the GBM invasion region. We longitudinally analyzed the vascular changes after blockade of VEGF signaling with DC101, an anti-mouse VEGFR2 monoclonal antibody, or IgG as a control (Fig. 3a). In mice treated with IgG, the luminal diameter of several vessels at the tumor periphery continuously increased by 2.6-fold (from 10 μm to 26 μm) over 84 h, equivalent to a 6.8-fold increase in the cross-sectional area. Importantly, DC101 treatment effectively suppressed vasodilation (Fig. 3b–d), indicating that VEGFR2 signaling is critical for the dynamic vascular remodeling associated with GBM invasion.

### Development of a Tie2-activating monoclonal antibody

**Fig. 4:**
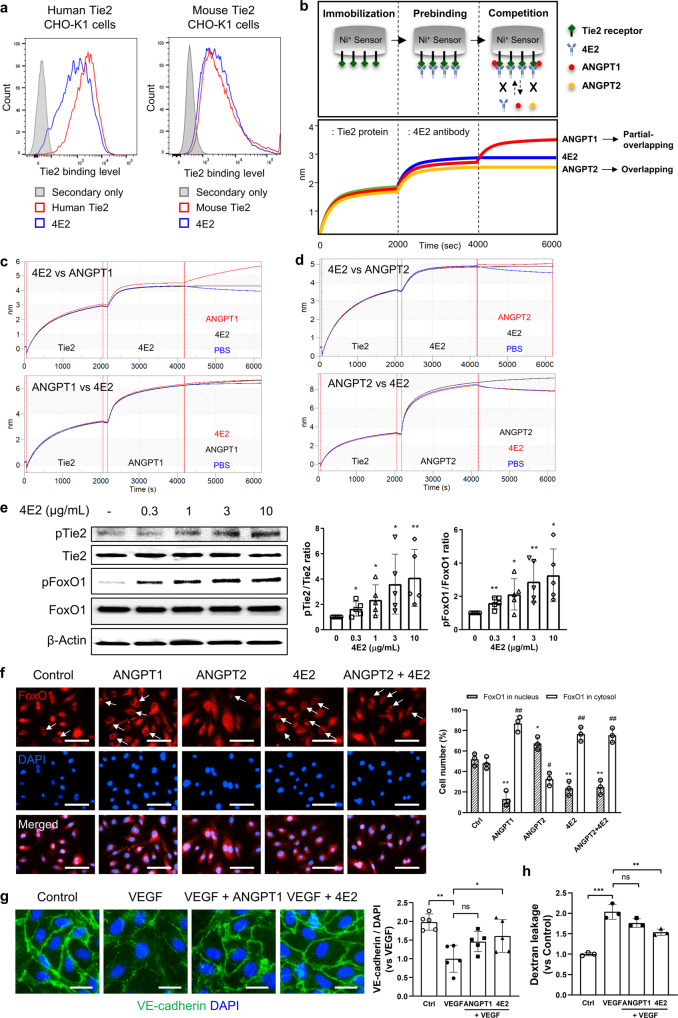
4E2 activates Tie2 signaling, similar to ANGPT1. **a** FACS plots showing the binding capacity of 4E2 to human (left) and mouse Tie2 receptors (right). **b** Outline of the OCTET competitive binding assay (upper) showing the binding of the 4E2 antibody to immobilized Tie2 followed by competitive binding with ANGPT1 and ANGPT2. Representative binding curves showing the competition modes of ANGPT1 and ANGPT2 with 4E2 (lower). **c**, **d** Binding curves showing the competitive binding of 4E2 with ANGPT1 (**c**) and ANGPT2 (**d**) to Tie2. **e** Immunoblots of phosphorylated Tie2 (pTie2) and FoxO1 (pFoxO1) in HUVECs treated with 4E2 and quantitative analysis (*n* = 5 per group). *, *P* < 0.05; **, *P* < 0.01 vs. 0 μg/mL 4E2. **f** Immunofluorescence images of FoxO1 in HUVECs and the proportions of its cytosolic and nuclear localization. The arrows indicate nuclear exclusion of FoxO1 (*n* = 3 per group). *, *P* < 0.05; **, *P* < 0.01 vs. Ctrl of nuclear FoxO1. #, *P* < 0.05; ##, *P* < 0.01 vs. Ctrl of cytosolic FoxO1. **g** Immunofluorescence images and quantification of junctional VE-cadherin in HUVECs (*n* = 5 per group). **h** Quantification of permeability in HUVECs. Scale bars: 100 µm (**f**), 25 µm (**g**). ns not significant; *, *P* < 0.05; **, *P* < 0.01; ***, *P* < 0.001.

Blockade of ANGPT2 effectively inhibits angiogenesis and vascular abnormalities in several tumor types by activating Tie2^25–27^. However, we were interested in developing alternative approaches for Tie2 activation because ANGPT2 is expressed locally in the GBM center. Therefore, we tried to develop an anti-Tie2 antibody with an agonistic function. From a fully human ScFv library, we selected a clone, 4E2, as a lead antibody due to its Tie2 binding activity. 4E2 has cross-reactivity for both human and mouse Tie2 (Fig. 4a), and its binding affinities (K_d_) for human and mouse Tie2 were 2.63 nM and 2.07 nM, respectively. Importantly, the binding assay showed that 4E2 can compete with ANGPT1 and ANGPT2 for binding to Tie2 (Fig. 4b–d), suggesting that the epitope of 4E2 overlaps with the ligand binding domain in Tie2.

From a functional perspective, treatment with 4E2 increased the phosphorylation of Tie2 and FoxO1, a downstream signaling molecule of Tie2, in human umbilical vein endothelial cells (HUVECs; Fig. 4e). 4E2 treatment induced cytosolic localization of FoxO1 similar to that driven by ANGPT1, an agonistic ligand of Tie2, and reversed the ANGPT2-driven nuclear localization of FoxO1 (Fig. 4f). In addition, 4E2 restored junctional VE-cadherin expression on VEGF-treated ECs and inhibited VEGF-induced dextran passage across an EC layer, similar to ANGPT1 (Fig. 4g, h). These data indicate that the 4E2 antibody with a high affinity for Tie2 can—similar to ANGPT1—activate Tie2 signaling and suggest that it could be used for animal studies due to its cross-reactivity for mouse Tie2.

### Antibody-based Tie2 activation rescues the vasculature in GBM, resembling the effects of VEGFR2 blockade

**Fig. 5:**
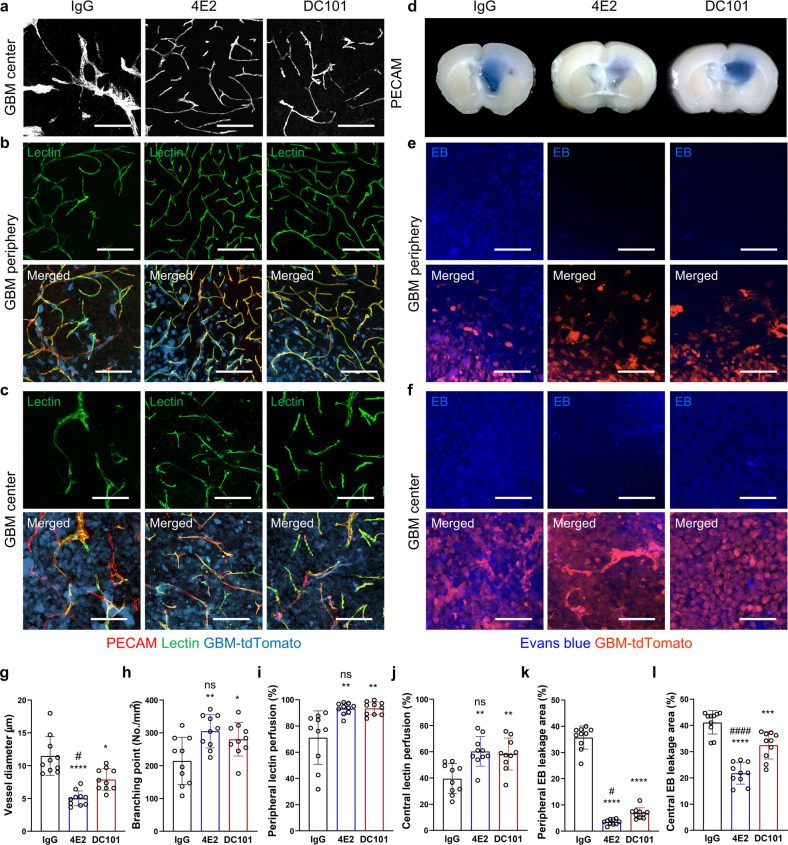
Antibody-based Tie2 activation and VEGFR2 blockade restore vascular morphology and function in GBM. Vascular changes in EGFR_VIII_ GBM 2 weeks after IgG, 4E2, or DC101 treatment. **a** PECAM immunostaining in the GBM center and quantification of vessel diameter (**g**, *n* = 10 per group) and vascular branching points (**h**, *n* = 10 per group). **b**, **c** Images of perfused lectin staining in the peripheral tumor vasculature (**b**), with quantification (**i**, *n* = 10 per group), and in the tumor center (**c**), with quantification (**j**, *n* = 10 per group). **d**–**f** Extravasated Evans blue (EB). **d** Coronal sections of GBM-bearing brain tissues. Immunofluorescence images showing EB leakage in the tumor periphery (**e**), with quantification (**k**, *n* = 10 per group) and in the tumor center (**f**), with quantification (**l**, *n* = 10 per group). Scale bars: 100 µm (**a**–**c**, **e**–**f**). *, *P* < 0.05; **, *P* < 0.01; ***, *P* < 0.001; ****, *P* < 0.0001 vs. IgG. ns not significant; #, *P* < 0.05; ####, *P* < 0.0001 vs. DC101.

To clarify whether antibody-based Tie2 activation and VEGFR2 blockade can normalize tumor vessels in spontaneous GBM models, we examined the morphology of EGFR_VIII_ GBM tumor vessels after treatment with IgG, 4E2, or DC101. Compared to treatment with the IgG control, treatment with 4E2 or DC101 significantly attenuated vasodilation and restored vascular branching (Fig. 5a, g, h). Treatment with 4E2 and DC101 also rescued the tight associations of ECs with pericytes and the BM, as shown in the magnified images (Supplementary Fig. 5a–d), although there was no discernable change visible in the wide-view images (Supplementary Fig. 5e, f). Regarding vascular function, compared to IgG treatment, treatment with 4E2 or DC101 increased the binding of infused lectin to ECs (Fig. 5b, c, i, j) and suppressed EB extravasation in the peripheral and central tumor regions (Fig. 5d–f, k–l). In comparison, 4E2 decreased EB leakage more potently than DC101. These findings indicate that antibody-based Tie2 activation and VEGFR2 inhibition can improve vascular morphology and function in spontaneous GBM models.

**Fig. 6:**
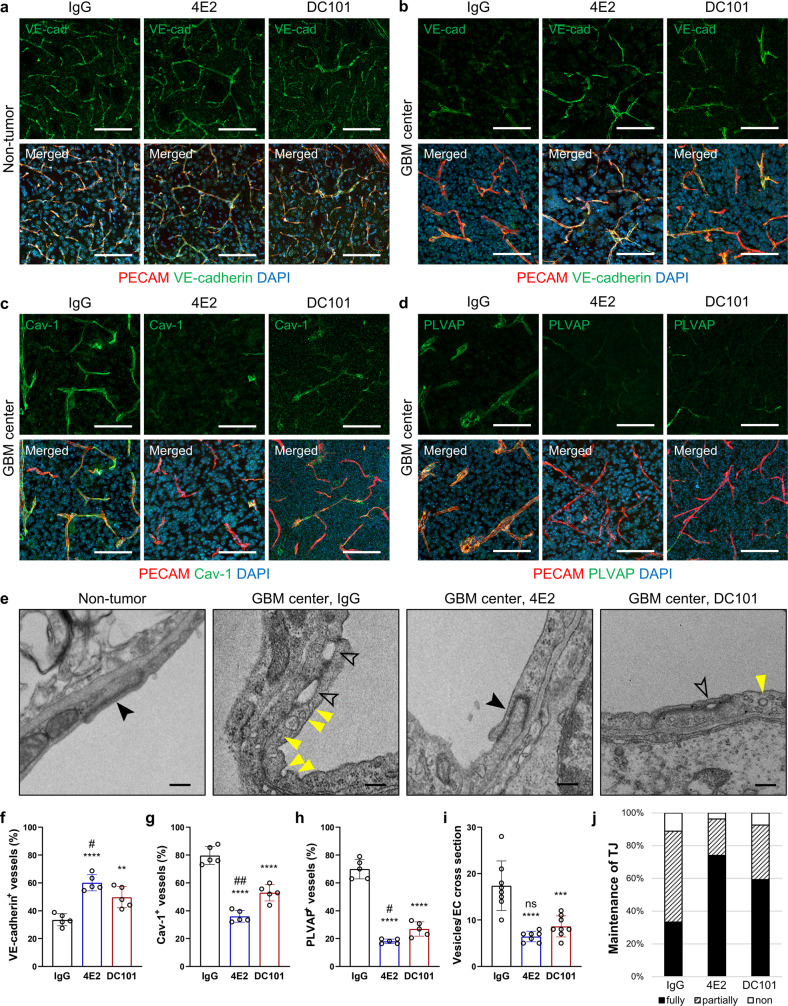
Antibody-based Tie2 activation and VEGFR2 blockade stabilize EC junctions and suppress EC transcytosis in GBM. Vascular assessment in EGFR_VIII_ GBMs at 2 weeks post-treatment with IgG, 4E2, or DC101. **a**, **b** Immunostaining for VE-cadherin in nontumor vessels (**a**) and GBM vessels (**b**), with quantification (**f**, *n* = 5 per group). **c** Immunostaining for Caveolin-1, a transcytosis marker, in tumor vessels, with quantification (**g**, *n* = 5 per group). **d** Immunostaining for PLVAP, a transcytosis marker, in tumor vessels, with quantification (**h**, *n* = 5 per group). **e** Transmission electron micrographs showing caveolar vesicle formation (yellow arrowheads) and cell-cell junctions in ECs of normal brain and GBM treated with IgG, 4E2, or DC101. The closed and open arrowheads indicate intact tight junctions and destabilized junctions, respectively. **i** Quantification of vesicles per EC cross-section (*n* ≥ 7 per group). **j** Proportion of intact tight junctions (TJs) (*n* = 27 per group). Scale bars: 100 µm (**a**-**d**), 200 nm (**e**). **, *P* < 0.01; ***, *P* < 0.001; ****, *P* < 0.0001 vs. IgG. ns not significant; #, *P* < 0.05; ####, *P* < 0.0001 vs. DC101.

To understand the mechanisms underlying the reduced vascular leakage in the presence of 4E2 and DC101, we examined the expression of markers associated with EC junction integrity and transcytosis in nontumor brain and EGFR_VIII_ GBM tissues after treatment with IgG, 4E2, and DC101. The expression of VE-cadherin was well maintained in nontumor vessels regardless of treatment. Although the level of VE-cadherin was greatly decreased in IgG-treated GBM vessels, it was restored by 4E2 or DC101 treatment (Fig. 6a, b, f). Transcytosis is suppressed in brain vessels. However, the transcytosis markers caveolin-1 (Cav-1) and PLVAP were robustly detected in IgG-treated GBM vessels, although their levels were substantially decreased by 4E2 or DC101 treatment (Fig. 6c, d, g, h). We further examined the ultrastructure of tumor ECs using transmission electron microscopy (TEM). IgG-treated tumor ECs exhibited increased vesicle formation and disrupted TJs compared to nontumor vessels. Treatment with 4E2 or DC101 decreased the number of vesicles and restored TJ integrity in tumor ECs (Fig. 6e, i, j). These findings suggest that both Tie2 activation and VEGFR2 inhibition restore the barrier function of GBM vessels by improving junctional integrity and limiting the number of caveolar vesicles.

To determine the effect of vascular normalization on tumor growth in the spontaneous GBM model, we monitored the in vivo bioluminescence emitted by tumor cells after treatment with 4E2, DC101, or IgG (Supplementary Fig. 6a). Although treatment with DC101 transiently delayed tumor growth for a week, the GBM tumors regrew, consistent with previous reports^28–30^. Treatment with 4E2 had no effect on GBM growth (Supplementary Fig. 6b, c). Because we observed infiltration of several types of immune cells, including CD8-positive T cells, in the GBM tissues (Supplementary Fig. 6d–g), we tested whether vessel normalization using 4E2 can improve the efficacy of immune checkpoint inhibition (Supplementary Fig. 6h). Treatment with 4E2 combined with an anti-PD-1 antibody significantly prolonged the survival of GBM-bearing mice compared to that of mice treated with the anti-PD-1 antibody alone (median survival time: 19 days with IgG, 21 days with 4E2, 30 days with the anti-PD-1 antibody, and 35 days with 4E2 plus the anti-PD-1 antibody; Supplementary Fig. 6i). Notably, three of the nine GBM-bearing mice treated with the combined therapy survived for longer than 40 days. These results indicate that vascular normalization can promote the therapeutic effects of anti-PD-1 immunotherapy on GBM.

### The agonistic anti-Tie2 antibody promotes VE-PTP-mediated VEGFR2 inhibition in GBM

**Fig. 7:**
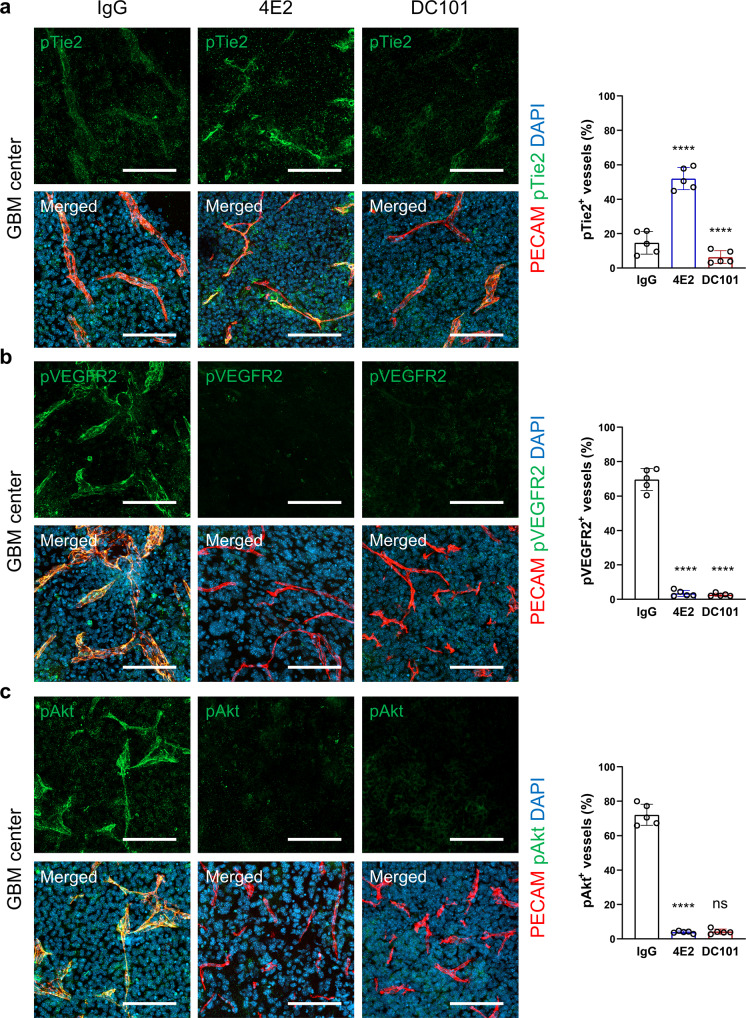
Tie2 activation reduces the level of phosphorylated VEGFR2 in GBM vessels. **a** Increase in phospho-Tie2 in GBM vessels by 4E2 treatment, with quantification (*n* = 5 per group). **b** Decrease in phospho-VEGFR2 in GBM vessels by 4E2 or DC101 treatment, with quantification (*n* = 5 per group). **c** Decrease in phospho-Akt in GBM vessels by 4E2 or DC101 treatment, with quantification (*n* = 5 per group). Scale bars: 100 µm (**a**–**c**). ns not significant; ****, *P* < 0.0001 vs. IgG.

To understand the mechanisms underlying the vascular normalization mediated by Tie2 activation and VEGFR2 blockade, we examined the activation of the corresponding downstream signaling molecules in GBM vessels after treatment with IgG, 4E2, or DC101. Treatment with 4E2 but not IgG or DC101 increased the level of phosphorylated Tie2 (pTie2) in both tumor vessels and nontumor vessels (Fig. 7a and Supplementary Fig. 7a, d). IgG-treated GBM vessels exhibited robust signals for phosphorylated VEGFR2 (pVEGFR2), which were markedly reversed by DC101 and, unexpectedly, by 4E2 (Fig. 7b). Nontumor vessels exhibited no noticeable signals in any experimental group (Supplementary Fig. 7b, e). Similarly, the level of phosphorylated Akt (pAkt), one of the major downstream molecules, was minimal in nontumor control vessels but was predominant in IgG-treated GBM vessels and substantially attenuated by 4E2 and DC101 (Fig. 7c). Although 4E2 treatment did not induce Akt phosphorylation in tumor vessels, it modestly increased the level of pAkt in nontumor brain vessels (Supplementary Fig. 7c, f), consistent with the role of Tie2 signaling in Akt phosphorylation in physiological contexts^31^. Our findings confirm that 4E2 can activate Tie2 signaling in vivo and suggest that this type of antibody-based Tie2 activation can attenuate vascular pathology at least partially by inhibiting VEGFR2 signaling in the context of GBM.

**Fig. 8:**
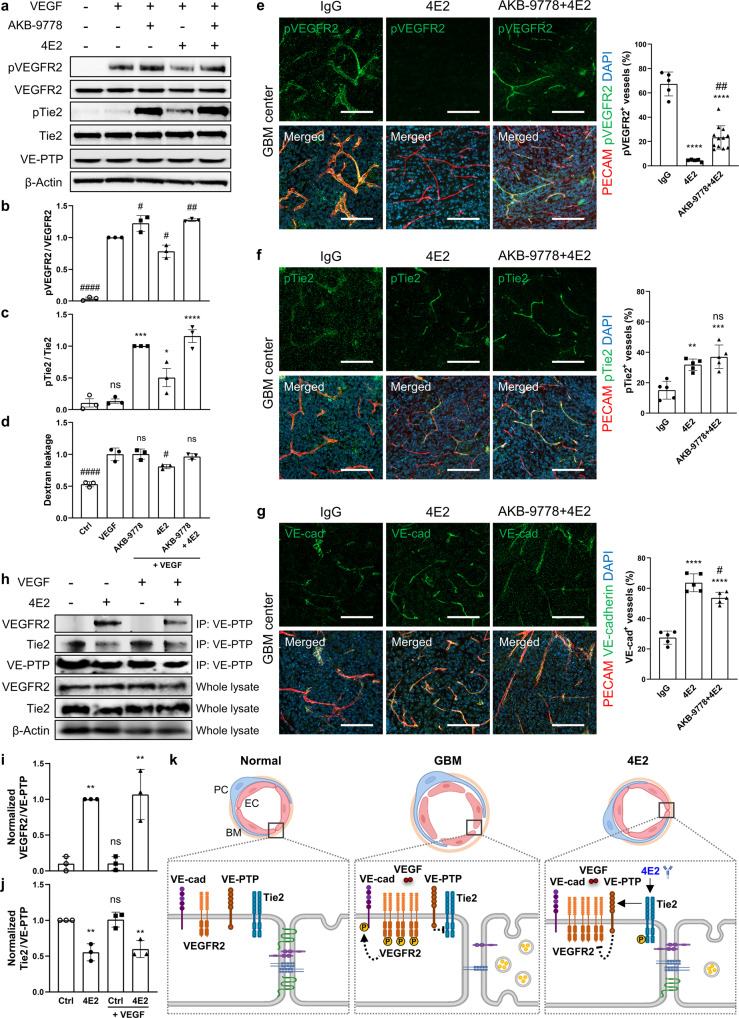
Antibody-based Tie2 activation attenuates VEGFR2 phosphorylation via VE-PTP. **a**–**c** Immunoblots (**a**) showing VE-PTP-mediated VEGFR2 dephosphorylation by 4E2 and quantification of the phospho-VEGFR2 (**b**, *n* = 3 per group) and phospho-Tie2 (**c**, *n* = 3 per group) levels. AKB-9778, inhibitor of VE-PTP. **d** Quantification of VEGF-induced dextran leakage in HUVECs (*n* = 3 per group). **e**–**g** Immunostaining and quantification of phospho-VEGFR2 (**e**), phospho-Tie2 (**f**), and VE-cadherin (**g**) in GBM vessels cotreated with 4E2 and AKB-9778 (*n* = 5 per group). **h** Increased immunoprecipitation of VE-PTP with VEGFR2 but not Tie2 by 4E2. **i**, **j** Quantification of the normalized VEGFR2/VE-PTP (**i**, *n* = 3 per group) and normalized Tie2/VE-PTP (**j**, *n* = 3 per group) levels. **k** Schematic showing the regulatory mechanisms of tumor vascular normalization by 4E2. EC, endothelial cell; PC, pericyte; BM, basement membrane. Scale bars: 100 µm (**e**–**g**). ns, not significant; *, *P* < 0.05; **, *P* < 0.01; ***, *P* < 0.001; ****, *P* < 0.0001 vs. Ctrl (**c**, **h**-**i**) or IgG (**e**-**g**). ns not significant; #, *P* < 0.05; ##, *P* < 0.01 vs. VEGF (**b**, **d**) or 4E2 (**e–g**).

Given that VE-PTP is an essential phosphatase for VEGFR2^32^, we explored whether VE-PTP is involved in the dephosphorylation of VEGFR2 induced by 4E2 treatment. We used AKB-9778, a small molecule inhibitor in phase 2 clinical trials for several ophthalmological diseases, to block VE-PTP expression in HUVECs^33^. Consistent with previous studies^32,34^, VEGF-induced VEGFR2 phosphorylation was slightly increased by AKB-9778 treatment. In contrast, 4E2 treatment suppressed VEGF-driven VEGFR2 phosphorylation, but cotreatment with AKB-9778 and 4E2 restored it to the level observed after AKB-9778 treatment alone (Fig. 8a, b). AKB-9778 also increased the pTie2 level regardless of 4E2 treatment (Fig. 8a, c), as reported previously^33,35^. Importantly, treatment with 4E2 alone but not in combination with ABK-9778 attenuated VEGF-induced dextran leakage though the EC layer (Fig. 8d), suggesting that VE-PTP could be involved in the effect of 4E2 on endothelial functions. Furthermore, we tried to validate the effect of AKB-9778 on GBM in vivo. Cotreatment with AKB-9778 significantly attenuated the 4E2-driven suppression of VEGFR2 phosphorylation but showed no additional effect on 4E2-induced Tie2 phosphorylation in GBM vessels (Fig. 8e, f), verifying that 4E2 can promote VE-PTP-mediated VEGFR2 dephosphorylation in the context of GBM. AKB-9778 moderately reduced the level of junctional VE-cadherin in 4E2-treated GBM vessels (Fig. 8g), suggesting that VE-PTP inhibition destabilizes endothelial junctions in normalized tumor vessels via VEGFR2 signaling.

Next, we investigated whether 4E2 can promote the formation of the VE-PTP–VEGFR2 complex by carrying out coimmunoprecipitation assays in HUVECs. 4E2 treatment markedly increased the coimmunoprecipitation of VEGFR2 with VE-PTP regardless of VEGF treatment, whereas it decreased the coimmunoprecipitation of Tie2 with VE-PTP (Fig. 8h–j). As VE-PTP was detected in both GBM vessels and nontumor brain vessels and its level was unaffected by treatment with either 4E2 or DC101 (Supplementary Fig. 7g), it is unlikely that the effect of 4E2 on reducing the pVEGFR2 level is mediated by VE-PTP upregulation. In addition, the VE-PTP level did not change in HUVECs after any treatment (Fig. 8a). As hypoxia-induced VEGF upregulation can activate VEGFR2, we analyzed HIF1-α expression in nontumor brain and GBM tissues after treatment with IgG, 4E2, or DC101. Compared to IgG treatment, 4E2 or DC101 treatment only modestly decreased HIF1-α immunoreactivity in GBM tissue (Supplementary Fig. 7h). These results suggest that 4E2 may reduce the pVEGFR2 level in hypoxic tumor tissue regardless of the VEGF levels. These results suggest that 4E2-triggered Tie2 activation can induce VEGFR2 dephosphorylation by increasing the formation of the VE-PTP–VEGFR2 complex (Fig. 8k).

## Discussion

Although orthotopic glioma models in mice have been useful for understanding the vascular abnormalities characterizing GBM, these models have limitations in faithfully reproducing the histopathological characteristics of grade IV glioma. By adopting somatic mutations frequently found in human GBM, we established a novel mouse model of spontaneous GBM characterized by heterogeneously disorganized vascular remodeling and impaired vascular functions. Importantly, this spontaneous GBM model was characterized by peripheral tumor invasion and an altered vasculature in the GBM infiltration zone, characteristics that have been poorly investigated^11^. In previous glioma models, tumor tissues are clearly demarcated from surrounding nontumor brain tissues, and the vascular morphology is distinct at the tumor-brain interface. In contrast, the tumor invasion region in this new GBM model is characterized by a gradual and progressive normal-to-tumor vascular transition. Therefore, this spontaneous GBM model provides insight into the vascular regulation closely intertwined with tumor invasion.

The VEGF/VEGFR2 and angiopoietin/Tie2 pathways are representative regulatory signaling systems involved in tumor vascular abnormalization^8,9,11^. In the present GBM model, the levels of VEGF and VEGFR2 increased gradually from the nontumor infiltrating area to the main tumor mass through the GBM infiltration region, showing a spatial correlation with the degree of vascular abnormalities. In addition, blockade of VEGFR2 signaling suppressed the dynamic vascular remodeling in the tumor peripheral region. Therefore, our findings identify VEGFR2 signaling as a key spatiotemporal system in the regulation of invasion-associated vascular remodeling in GBM. On the other hand, the vascular Tie2 level was relatively constant in both brain and tumor tissues, and ANGPT2 expression was restricted to a portion of vessels in the GBM center. Based on this expression pattern, it is unlikely that ANGPT2 and Tie2 are related to the normal-to-tumor vascular changes in the GBM invasion region. Increased Tie2 expression in the GBM center may be stimulated by hypoxia or proinflammatory cytokines^36^. Therefore, a strategy based on blockade of ANGPT2 needs to be supplemented for better vascular normalization in GBM^12^. In addition, the constant Tie2 level throughout nontumor and tumor vessels may be consistent with the decreased vascular sprouting observed in our GBM models, considering that Tie2 expression can be downregulated in angiogenic ECs^27^.

In accordance with this strategic demand, we developed a novel agonistic antibody, 4E2, to activate Tie2 regardless of the availability of ANGPT2, although there is an anti-ANGPT2 antibody that can activate Tie2 in complex with an antigen. This new Tie2-activating antibody effectively restored vascular morphology and function in both the tumor periphery and center, similar to the effects of the anti-VEGFR2 blocking antibody DC101. Interestingly, the 4E2 antibody appeared to induce tumor vascular normalization by a dual action mechanism: Tie2 activation and VEGFR2 inhibition. However, the mechanism by which Tie2 activation can lead to VEGFR2 inhibition remains to be determined. According to an earlier report, Tie2 activation via ANGPT1 promoted mDia-Src complex formation, thereby inactivating VEGFR2, in a VEGF-induced vascular leakage model^8^. In other studies, VEGFR2 was dephosphorylated by VE-PTP in a Tie2-dependent manner, leading to decreased phosphorylation of VE-cadherin during vascular morphogenesis^32,34^. Our findings based on pharmacological inhibition and coimmunoprecipitation of VE-PTP suggest that Tie2 activation by the 4E2 antibody can induce the interaction of VE-PTP with VEGFR2, followed by VE-PTP-mediated VEGFR2 inhibition, in a pathological context. The AKB-9778-driven VE-cadherin suppression, although moderate, in 4E2-treated GBM vessels suggests that VE-PTP inhibition might offset the normalization of tumor vessels induced by Tie2 activation, in line with the complicated effect of VE-PTP inhibition on the integrity of EC junctions^35^. Attenuation of persistent Akt phosphorylation may also be involved in GBM vessel normalization, because chronic or excessive Akt activation is implicated in vascular pathologies^37,38^. Moreover, 4E2 restored vascular morphology and function more effectively than DC101. Additional mechanisms of action of 4E2 may underlie its more beneficial effects, considering that 4E2 increased the phosphorylation of FoxO1, which is one of the biochemical changes resulting from Tie2 activation that is implicated in vessel remodeling^39^. Therefore, agonistic antibody-based Tie2 activation could be applicable to various diseases with vascular leakage.

Antiangiogenic approaches have shown small or only transient effects on tumor growth and have been developed into combination therapies based on vascular normalization^1,40^. Although vessel normalization mediated by blockade of VEGF or ANGPT2 increased the efficacy of chemotherapy in mouse GBM models^12,41^, it improved the quality of life but not overall survival in human trials^14,16^. Recently, immunotherapy has been shown to be successful in several types of cancer, but it showed limited effects in GBM as monotherapy^42,43^. Therefore, there have been several attempts to test the combined effect of vascular normalization therapy and immunotherapy on GBM growth in experimental animal models^44,45^. However, these preclinical studies used orthotopic or xenograft models in immunodeficient animals and need to be complemented to develop more clinically relevant strategies. In the present study based on a model of spontaneous GBM with invasive traits in immunocompetent mice, vessel normalization by 4E2 improved the therapeutic efficacy of anti-PD-1 treatment, as shown by the increased median survival rate and the presence of strong responders with notably prolonged survival. In line with the clinical trials in GBM patients^46,47^, our findings suggest a novel approach of vascular normalization to increase the effectiveness of GBM immunotherapy.

Vasodilation in the brain has been highlighted in neurovascular (NV) coupling, as well as in the present GBM model. This dynamic vascular change is commonly triggered by external stimuli, such as neural activation in NV coupling and tumor cell infiltration in tumor vasodilation. However, vascular dilation has context-dependent properties. NV coupling is a highly dynamic process; vasodilation occurs in an eNOS-dependent manner within a range of milliseconds in response to nearby neural activity^24,48,49^, and enlarged vessels then return to a normal state immediately after the neuron becomes quiescent. Therefore, vasodilation associated with NV coupling is a physiological change. In contrast, brain vessels were found to undergo continuous and irreversible enlargement over several days, which was initiated by a small cluster of infiltrating GBM cells and dependent on VEGF signaling, resulting in pathological vascular remodeling. Tumor vessels were also found to be increasingly dilated in deeper tumor specimens compared to peripheral specimens from patients with GBM, suggesting the clinical relevance of tumor vasodilation. Thus, the tumor-vascular interaction in GBM is a slow and unidirectional process, in contrast to the highly dynamic and reversible process of NV coupling.

Although tumor angiogenesis has long been considered a major vascular change in GBM^13,50^, there was no clear angiogenic signature in this new GBM model. The findings in the present study revealed previously unknown invasion-associated vascular changes. First, we characterized the progressive normal-to-tumor vascular transition in the GBM infiltrating region outside the main tumor mass, pointing to a spatial pattern of vascular heterogeneity in GBM. Treatment with a novel agonistic anti-Tie2 antibody, 4E2, resulted in vascular normalization throughout the GBM tissues, including the tumor periphery and tumor center, thereby improving the efficacy of combined immune checkpoint therapy. Mechanistically, antibody-based Tie2 activation induces VE-PTP-mediated VEGFR2 inhibition in tumor vessels in a ligand-independent manner, suggesting a potential strategy based on multiple mechanisms of action for regulating GBM vessels.

## Supplementary information

Supplementary information

## References

[1] Carmeliet P, Jain RK (2011). Principles and mechanisms of vessel normalization for cancer and other angiogenic diseases. Nat. Rev. Drug Discov..

[2] De Palma M, Biziato D, Petrova TV (2017). Microenvironmental regulation of tumour angiogenesis. Nat. Rev. Cancer.

[3] Maes H (2014). Tumor vessel normalization by chloroquine independent of autophagy. Cancer Cell.

[4] Broekman ML (2018). Multidimensional communication in the microenvirons of glioblastoma. Nat. Rev. Neurol..

[5] Das S, Marsden PA (2013). Angiogenesis in glioblastoma. N. Engl. J. Med..

[6] Hambardzumyan D, Bergers G (2015). Glioblastoma: Defining tumor niches. Trends Cancer.

[7] Chae JK (2000). Coadministration of angiopoietin-1 and vascular endothelial growth factor enhances collateral vascularization. Arterioscler. Thromb. Vasc. Biol..

[8] Gavard J, Patel V, Gutkind JS (2008). Angiopoietin-1 prevents VEGF-induced endothelial permeability by sequestering Src through mDia. Dev. Cell..

[9] Winkler F (2004). Kinetics of vascular normalization by VEGFR2 blockade governs brain tumor response to radiation: role of oxygenation, angiopoietin-1, and matrix metalloproteinases. Cancer Cell.

[10] Piao Y (2016). Novel MET/TIE2/VEGFR2 inhibitor altiratinib inhibits tumor growth and invasiveness in bevacizumab-resistant glioblastoma mouse models. Neuro. Oncol..

[11] Kim IK (2018). Sox7 promotes high-grade glioma by increasing VEGFR2-mediated vascular abnormality. J. Exp. Med.

[12] Park JS (2016). Normalization of tumor vessels by Tie2 activation and Ang2 inhibition enhances drug delivery and produces a favorable tumor microenvironment. Cancer Cell.

[13] Cohen MH, Shen YL, Keegan P, Pazdur R (2009). FDA drug approval summary: bevacizumab (Avastin) as treatment of recurrent glioblastoma multiforme. Oncologist.

[14] Gilbert MR (2014). A randomized trial of bevacizumab for newly diagnosed glioblastoma. N. Engl. J. Med..

[15] Leow CC (2012). MEDI3617, a human anti-angiopoietin 2 monoclonal antibody, inhibits angiogenesis and tumor growth in human tumor xenograft models. Int. J. Oncol..

[16] Lee EQ (2020). NRG/RTOG 1122: A phase 2, double-blinded, placebo-controlled study of bevacizumab with and without trebananib in patients with recurrent glioblastoma or gliosarcoma. Cancer.

[17] Lee JH (2018). Human glioblastoma arises from subventricular zone cells with low-level driver mutations. Nature.

[18] Li G, Sachdev U, Peters K, Liang X, Lotze MT (2019). The VE-PTP inhibitor AKB-9778 improves antitumor activity and diminishes the toxicity of interleukin 2 (IL-2) administration. J. Immunother..

[19] Nawroth R (2002). VE-PTP and VE-cadherin ectodomains interact to facilitate regulation of phosphorylation and cell contacts. EMBO J..

[20] Baumer S (2006). Vascular endothelial cell-specific phosphotyrosine phosphatase (VE-PTP) activity is required for blood vessel development. Blood.

[21] Huszthy PC (2012). In vivo models of primary brain tumors: pitfalls and perspectives. Neuro. Oncol..

[22] McDermott DF (2018). Clinical activity and molecular correlates of response to atezolizumab alone or in combination with bevacizumab versus sunitinib in renal cell carcinoma. Nat. Med..

[23] Jain RK (2014). Antiangiogenesis strategies revisited: from starving tumors to alleviating hypoxia. Cancer Cell.

[24] Metea MR, Newman EA (2006). Glial cells dilate and constrict blood vessels: a mechanism of neurovascular coupling. J. Neurosci..

[25] Chae SS (2010). Angiopoietin-2 interferes with anti-VEGFR2-induced vessel normalization and survival benefit in mice bearing gliomas. Clin. Cancer Res.

[26] Wick W (2017). Lomustine and bevacizumab in progressive glioblastoma. N. Engl. J. Med..

[27] Felcht M (2012). Angiopoietin-2 differentially regulates angiogenesis through TIE2 and integrin signaling. J. Clin. Invest..

[28] Keunen O (2011). Anti-VEGF treatment reduces blood supply and increases tumor cell invasion in glioblastoma. Proc. Natl Acad. Sci. USA..

[29] Lamszus K, Kunkel P, Westphal M (2003). Invasion as limitation to anti-angiogenic glioma therapy. Acta Neurochir. Suppl..

[30] Narayana A (2009). Antiangiogenic therapy using bevacizumab in recurrent high-grade glioma: impact on local control and patient survival. J. Neurosurg..

[31] Kontos CD (1998). Tyrosine 1101 of Tie2 is the major site of association of p85 and is required for activation of phosphatidylinositol 3-kinase and Akt. Mol. Cell Biol..

[32] Hayashi M (2013). VE-PTP regulates VEGFR2 activity in stalk cells to establish endothelial cell polarity and lumen formation. Nat. Commun..

[33] Shen J (2014). Targeting VE-PTP activates TIE2 and stabilizes the ocular vasculature. J. Clin. Invest..

[34] Mellberg S (2009). Transcriptional profiling reveals a critical role for tyrosine phosphatase VE-PTP in regulation of VEGFR2 activity and endothelial cell morphogenesis. Faseb. J..

[35] Frye M (2015). Interfering with VE-PTP stabilizes endothelial junctions in vivo via Tie-2 in the absence of VE-cadherin. J. Exp. Med.

[36] Willam C (2000). Tie2 receptor expression is stimulated by hypoxia and proinflammatory cytokines in human endothelial cells. Circ. Res..

[37] Phung TL (2006). Pathological angiogenesis is induced by sustained Akt signaling and inhibited by rapamycin. Cancer Cell.

[38] Cui Y (2021). Brain endothelial PTEN/AKT/NEDD4-2/MFSD2A axis regulates blood-brain barrier permeability. Cell Rep..

[39] Kim M (2016). Opposing actions of angiopoietin-2 on Tie2 signaling and FOXO1 activation. J. Clin. Invest.

[40] Alphandery E (2018). Glioblastoma treatments: An account of recent industrial developments. Front. Pharmacol..

[41] Dickson PV (2007). Bevacizumab-induced transient remodeling of the vasculature in neuroblastoma xenografts results in improved delivery and efficacy of systemically administered chemotherapy. Clin. Cancer Res.

[42] Reiss SN, Yerram P, Modelevsky L, Grommes C (2017). Retrospective review of safety and efficacy of programmed cell death-1 inhibitors in refractory high grade gliomas. J. Immunother. Cancer.

[43] Kurz SC (2018). PD-1 inhibition has only limited clinical benefit in patients with recurrent high-grade glioma. Neurology.

[44] Di Tacchio M (2019). Tumor vessel normalization, immunostimulatory reprogramming, and improved survival in glioblastoma with combined inhibition of PD-1, angiopoietin-2, and VEGF. Cancer Immunol. Res.

[45] Scholz A (2016). Endothelial cell-derived angiopoietin-2 is a therapeutic target in treatment-naive and bevacizumab-resistant glioblastoma. EMBO Mol. Med..

[46] Nayak L (2021). Randomized phase II and biomarker study of pembrolizumab plus bevacizumab versus pembrolizumab alone for patients with recurrent glioblastoma. Clin. Cancer Res.

[47] Ho RLY, Ho IAW (2021). Recent advances in glioma therapy: Combining vascular normalization and immune checkpoint blockade. Cancers (Basel).

[48] Iadecola C (2017). The neurovascular unit coming of age: A journey through neurovascular coupling in health and disease. Neuron.

[49] Attwell D (2010). Glial and neuronal control of brain blood flow. Nature.

[50] Labussiere M (2016). Angiopoietin-2 may be involved in the resistance to bevacizumab in recurrent glioblastoma. Cancer Invest.

