# Supplementary material for: An agonistic anti-Tie2 antibody suppresses the normal-to-tumor vascular transition in the glioblastoma invasion zone
Source: Exp Mol Med. 2023 Feb 24;55(2):470–84. doi: 10.1038/s12276-023-00939-9 (PMC9981882; doi:10.1038/s12276-023-00939-9)
Supplement: Supplementary file 1 — Supplementary information [file 12276_2023_939_MOESM1_ESM.pdf]

Supplementary information

Supplemental methods

Orthotopic GL261 glioma model

Mouse GL261 glioma cell line was a generous gift from Dr. Karl Plate (Goethe University Medical School, Germany), with which GL261-GFP cell line was created by lentiviral infection of GFP-expressing vector into the GL261 cells. Under anesthesia, 2.0 x 10^5 of GL261 or GL261-GFP mouse glioma cells were implanted into right hemisphere of 8 to 10-week-old C57BL/6J mice with the aid of stereotaxic apparatus (RWD Life Science, San Diego).

SPR measurement of 4E2

The binding kinetics of 4E2 was measured with SPR (Bioacore T200, Cytiva, Marlborough). 8 nM of antibody was immobilized with protein-A chip with a 20 ul/min for 30 sec. For association, human or mouse Tie2 (Sino Biologics) in PBS containing 0.005 % Tween 20 was flowed with a 30 μl/min for 5 min. The concentration of analyte was 0.781, 1.563, 3.125, 6.25, 12.5, 50, 100, 200 and 400 nM. Dissociation was done with the same buffer with a 30 μl/min for 10 min. 10 mM Glycine buffer (pH 1.5) was used for the regeneration. The association and dissociation constant were evaluated with BIAevaluation software version 3.0 with a 1:1 stoichiometry.

Permeability assay

HUVECs were grown as mature monolayers (6.5×10^4 cells/well) for 3 days on 0.1% gelatin-coated 0.4 μm-pore inserts (PIHP01250, Merck Millipore). Cells were treated with 100 ng/ml angiopoietin-1 (ANGPT1, 923-AN/CF, R&D Systems), 30 μg/ml 4E2, or 5 μM AKB-9778 (HY-109041, MedChemExpress) for 30 min, followed by post-treatment with human VEGF-165 protein (293-VE/CF, R&D Systems) for 150 min. Then 70 kDa FITC-dextran (10 mg/ml, FD70S, Sigma-Aldrich) was incubated in the upper chamber for 30 min. The amount of FITC-dextran in each sample from the bottom chamber was measured using the Fluorescence Microplate Reader (Chameleon, Hidex).

Flow cytometry

For target binding, 1x10^5 cells of human Tie2-overexpressing CHO-K1 cells were washed at room temperature in PBS containing 10% fetal bovine serum (PBS/FBS) and incubated with 4E2 at 4 μg/ml final concentration for 30 min at 4 ℃. Cells were stained with PE-conjugated anti-human FC-antibody in PBS/FBS at 4 °C for 15 min. As mentioned above, mouse Tie2-overexpressed CHO-K1 cells were incubated with 10 μg/ml of 4E2 for 30 min at 4 ℃. For positive controls, 4 μg/ml of PE-conjugated anti-human and anti-mouse Tie2 antibodies were bound to human and mouse Tie2-overexpressing CHO-K1 cells. After washing in ice-cold PBS/FBS, the stained cells were resuspended in PBS/FBS and kept on ice for FACS. For competitive binding assay, human Tie2-overexpressing CHO-K1 cells were allowed to bind 10 μg/ml of 4E2 for 30 min, and then bind 0-30 μg/ml His-clustering ANGPT1 (or ANGPT2) for 15 min. 4E2 and ANGPTs are detected by fluorescently labeled-secondary antibodies, respectively. The stained cells were analyzed using a BD FACS Lyric.

Bioluminescence imaging

Prior to imaging, the mice bearing brain tumors were anesthetized with inhalation of isoflurane gas, and injected intravenously with 150 mg/kg of D-luciferin solution (LUCK-100, Gold Biotechnology, St. Louis). Luminescence signal emitted from tumors was captured 10 min after injection using an IVIS Spectrum (IVIS Lumina S5, PerkinElmer, Waltham). *In vivo* bioluminescence imaging was acquired using 10–60 seconds exposure and small or large binning depending on the light produced, 1 f/stop, and an open filter. Living image software (Caliper Life Sciences) was used for all image acquisition and data analysis.

T2-weighted MRI imaging

Mice were initially anesthetized with 4% isoflurane (Piramal Critical Care, Bethlehem) in a 7:1 mixture of air and oxygen. The anesthesia level was reduced to 1.5% during the data acquisition. The head of the animal was fixed with a bite bar to reduce the head motion. The respiration rate of the animal was monitored using an MRI- compatible pressure-sensitive pillow (Model 1025, SA Instruments, Stony Brook) and maintained within a normal range of 60-90 bpm. The body temperature was monitored using a rectal probe (Model 1025, SA Instruments) and maintained at 37 ± 0.5 ℃ by using a hot air. MRI experiments were performed on a 3-Tesla MRS 3000 preclinical scanner of 17-cm bore size (MR solutions, Guildford) with a 20-mm birdcage mouse head coil. For MR imaging, a T2-weighted 2D Fast spin echo (FSE) sequence was used, and the scan parameters were as follows: scan direction =

axial, TR = 3,000 ms, TE = 68 ms, TE_{base}/TE_{eff} = 17 ms/68 ms, echo train length = 8, number of averages = 8, field of view = 22 x 22 mm^2, matrix size = 256×256, slice thickness = 0.8 mm (no gap), number of slices = 16, and total scan time = 11 min 4 sec.

Custom-built confocal microscopy for intravital imaging

To visualize *in vivo* vascular remodeling, a custom-built video-rate laser-scanning confocal microscopy system was used as described[1]. The imaging system was equipped with three continuous wave laser modules with output wavelengths at 488 nm (MLD488, Cobolt AB, Solna), 561 nm (Jive, Cobolt AB), and 640 nm (MLD640, Cobolt AB), respectively. Combining a rapidly rotating 36 facets polygonal mirror (MC-5, aluminum coated, Lincoln Laser, Phoenix) and galvanometer mirror scanner (6230H, Cambridge Technology, Bedford), a video-rate Raster-pattern laser-scanning was achieved. The scanning laser beam was then delivered to the deep brain tissue through the tube imaging window by using objective lenses PlanApoλ 10x (Nikon, 10×, 0.45NA). The multi-color fluorescence signals were split by dichroic beam splitters (FF484-Di01, FF560-Di01, FF649-Di01, Semrock, West Henrietta) and detected by photomultiplier tubes (PMT; R9110, Hamamatsu) through bandpass filters (FF02-525/50, FF01-600/37, FF01-685/40, Semrock). Electronic signal outputs from the PMTs were digitized by a frame grabber (Solios, Matrox). The video-rate images with a frame rate of 15 Hz and a size of 1024 × 1024 pixels were displayed and recorded in real-time by custom-written software using Matrox Imaging Library (MIL10, Matrox).

Window implantation and intravital imaging

To monitor tumor microenvironment in deep brain region, we developed a customized brain-implantable tube imaging window chamber integrated with custom-built laser-scanning confocal microscope. After craniotomy with diameter of 3 mm, the cortical tissue was carefully aspirated until the tumor-initiating area appeared. Then the tube window chamber, consisting of a stainless-steel cannula (height: 1.8mm, OD: 3.2 mm, ID: 2.8mm) and a coverglass (0.1mm thickness, 3mm diameter; Warner Instruments, Holliston), was gently implanted. The top part of the window chamber and headplate was tightly glued to the skull surface using light-cured dental adhesive (Optibond FL, Kerr, Brea) and dental acrylic resin. To prevent the window surface from contamination, the implanted tube imaging window chamber was filled with a low viscosity silicone elastomer (Kwik-Cast sealant, World Precision Instruments, Sarasota) during the recovery period, which was then removed right before the intravital imaging. For

intravital imaging, the mouse was anesthetized and placed on a xyz-motorized stage with a customized stereotaxic device. To visualize the blood vessels, Dylight-488 conjugated Lycopersicum esculentum lectin (DL-1174, Vector) was intravenously injected before the intravital imaging.

Image processing and data analysis of intravital imaging

To enhance the signal-to-noise ratio of fluorescence signal from the tumor microenvironment, 60 frames (4 seconds with 15 frames/sec) were recorded and then averaged to generate a single image while removing the motion artifact using a custom-written MATLAB program. High-resolution z-stack images (with 10 μm-interval for wide-view and 3 μm-interval for magnified-view) were acquired and a maximum intensity projection of z-stack images was generated using Image J (NIH). The quantification of 2D-projection images was conducted using a commercial image analysis software, IMARIS 9.0.2 (Bitplane); measurement function was used for vessel diameter analysis.

Western blot

HUVECs were washed with PBS and homogenized in RIPA buffer (Thermo Fisher), and the protein concentration was determined using a BCA protein assay kit (Thermo Fisher). For each sample, 20 μg of protein was loaded into 4–12% NuPage Bis-Tris gels (Invitrogen) and transferred to a PVDF membrane (Invitrogen). The blocked membrane was then incubated with antibodies for pVEGFR2 (2478, Cell signaling Technology), VEGFR2 (2479, Cell signaling Technology), VE-cadherin (2500, Cell signaling Technology), pFoxO1 (9461S, Cell signaling Technology), FoxO1 (2880, Cell signaling Technology), Tie2 (7403, Cell signaling Technology), pTie2 (AF2720, R&D Systems), VE-PTP[19], and β-actin (SC-47778, Santa Cruz Biotechnology). The immunoreactive bands were visualized with a chemiluminescent reagent kit (Pierce Chemical, Dallas).

Transmission electron microscopy (TEM)

Mice were cardiac perfused with 2% PFA and 2% glutaraldehyde in PBS. Brain was minced into small pieces (less than 1 mm^3) and incubated in the same fixative overnight at 4 °C. Fixed brain was post-fixed with 1% osmium tetroxide in PBS at 4 °C for 1 hr. Brain was dehydrated with a series of ethanol gradient, substituted with propylene oxide, and finally embedded in EMbed-812 resin (EMS). Polymerization was performed at 60 °C for 40 hr. Brain resin block

was cut at a thickness of 70 nm (Leica EM UC7, Leica Biosystems). Sections were stained with 2.5% uranyl acetate and Reynold's lead citrate. Images were viewed with Tecnai G2 spirit TWIN (FEI, Hillsboro) electron microscope at 120 kV acceleration voltage.

Human brain tumor specimens

After receiving informed consent, tumor specimens with corresponding clinical records were obtained from patients undergoing surgery at the Samsung Medical Center. This study was approved by the local institutional review board (IRB file number 201004004) and conducted in accordance with all relevant ethical regulations for research using human specimens. To obtain non-tumor brain samples, the local institutional review board (IRB file number 906-210118-BR-004-01) of Chonnam National University Medical School and Hospital approved all autopsies as research activity involving non-living, post-mortem subjects in accordance with ethical regulations and waived the requirement to obtain informed consent owing to the inaccessibility to and unavailability of personal identification information and testing for heritable traits of the deceased. Accordingly, all specimens and data, with the exception of age, gender, and medical histories, were anonymized prior to all analyses.

Supplemental reference

1 Lee, J., Kong, E., Hong, S., Moon, J. & Kim, P. In vivo longitudinal visualization of the brain neuroinflammatory response at the cellular level in LysM-GFP mice induced by 3-nitropropionic acid. *Biomed. Opt. Express* **11**, 4835-4847 (2020).

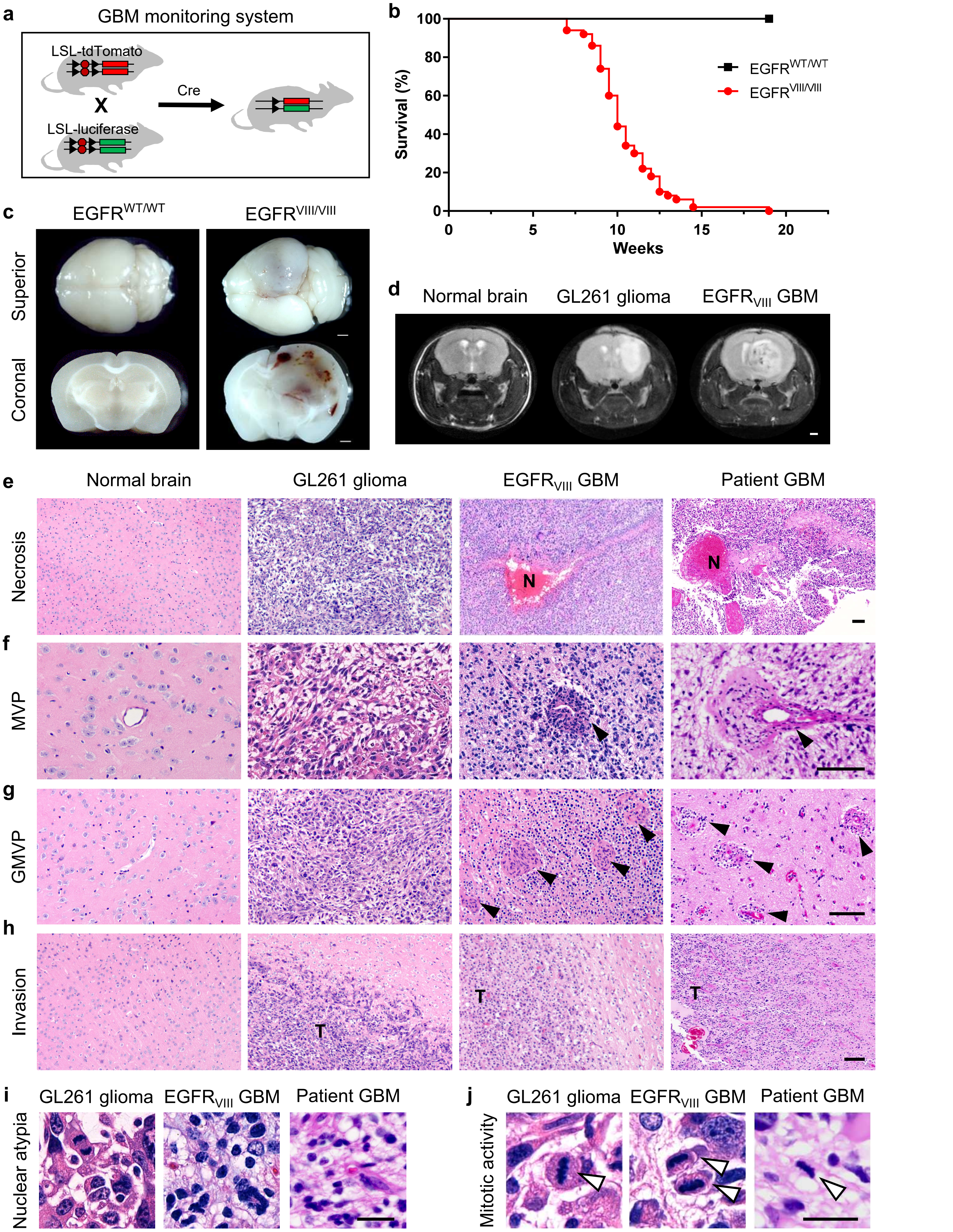
a
GBM monitoring system
LSL-tdTomato
X
LSL-luciferase
Cre
b
Survival (%)
100
80
60
40
20
0
0
5
10
15
20
Weeks
EGFR^WT/WT
EGFR^VIII/VIII
c
EGFR^WT/WT
EGFR^VIII/VIII
Superior
Coronal
d
Normal brain
GL261 glioma
EGFR_VIII GBM
e
Normal brain
GL261 glioma
EGFR_VIII GBM
Patient GBM
Necrosis
N
N
f
MVP
g
GMVP
h
Invasion
T
T
T
i
GL261 glioma
EGFR_VIII GBM
Patient GBM
Nuclear atypia
j
GL261 glioma
EGFR_VIII GBM
Patient GBM
Mitotic activity

Supplementary Fig. 2

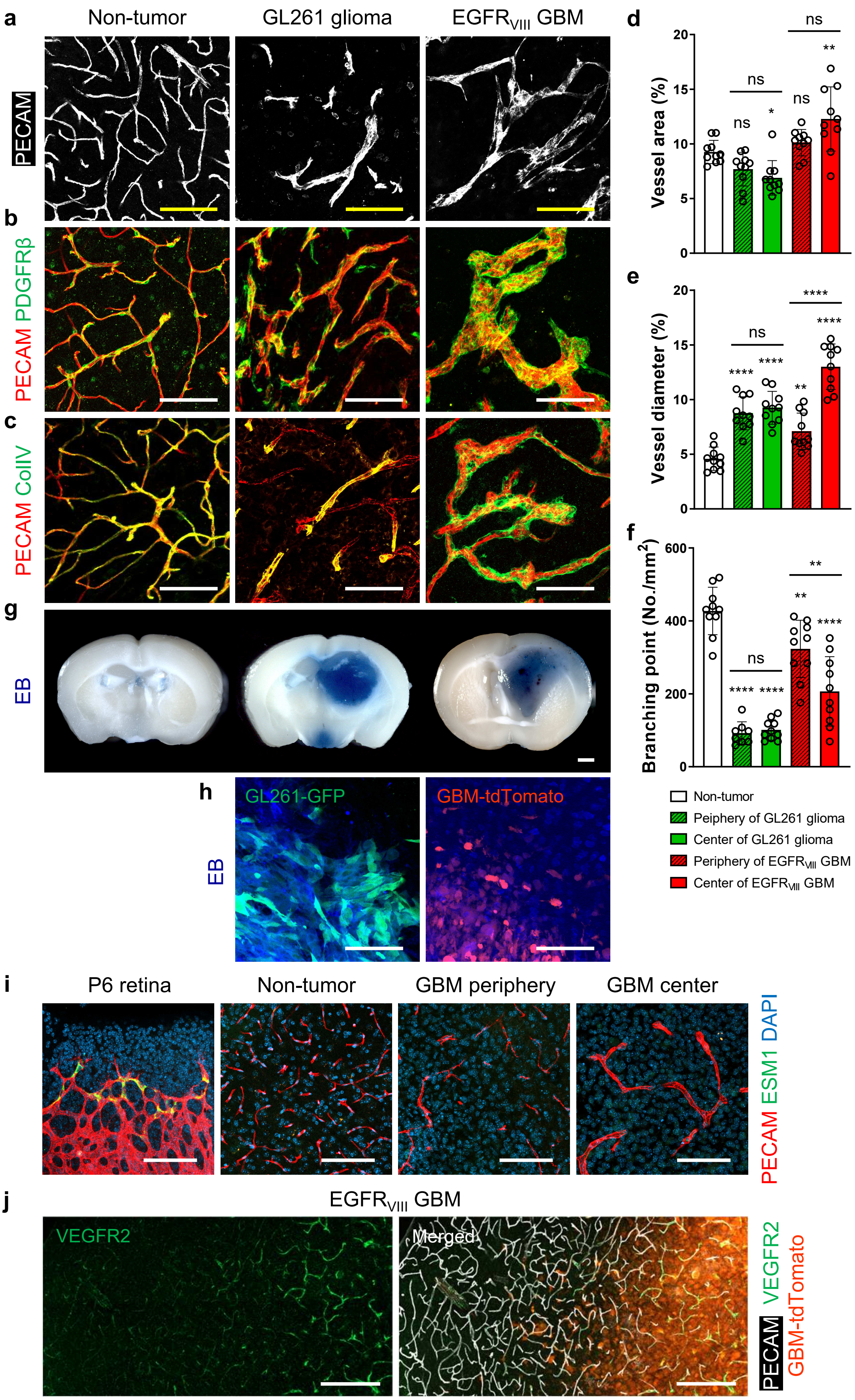

Supplementary Fig. 3

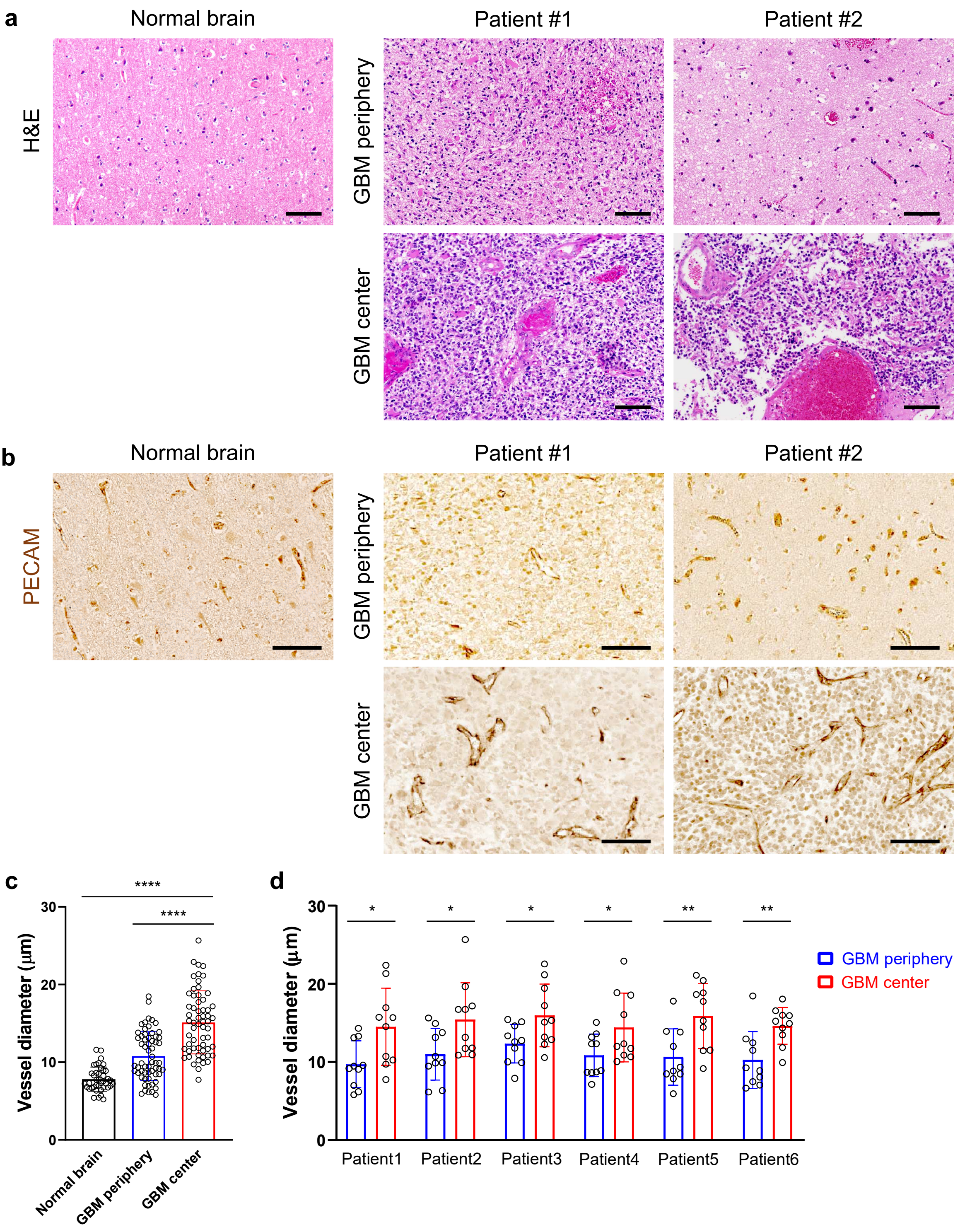

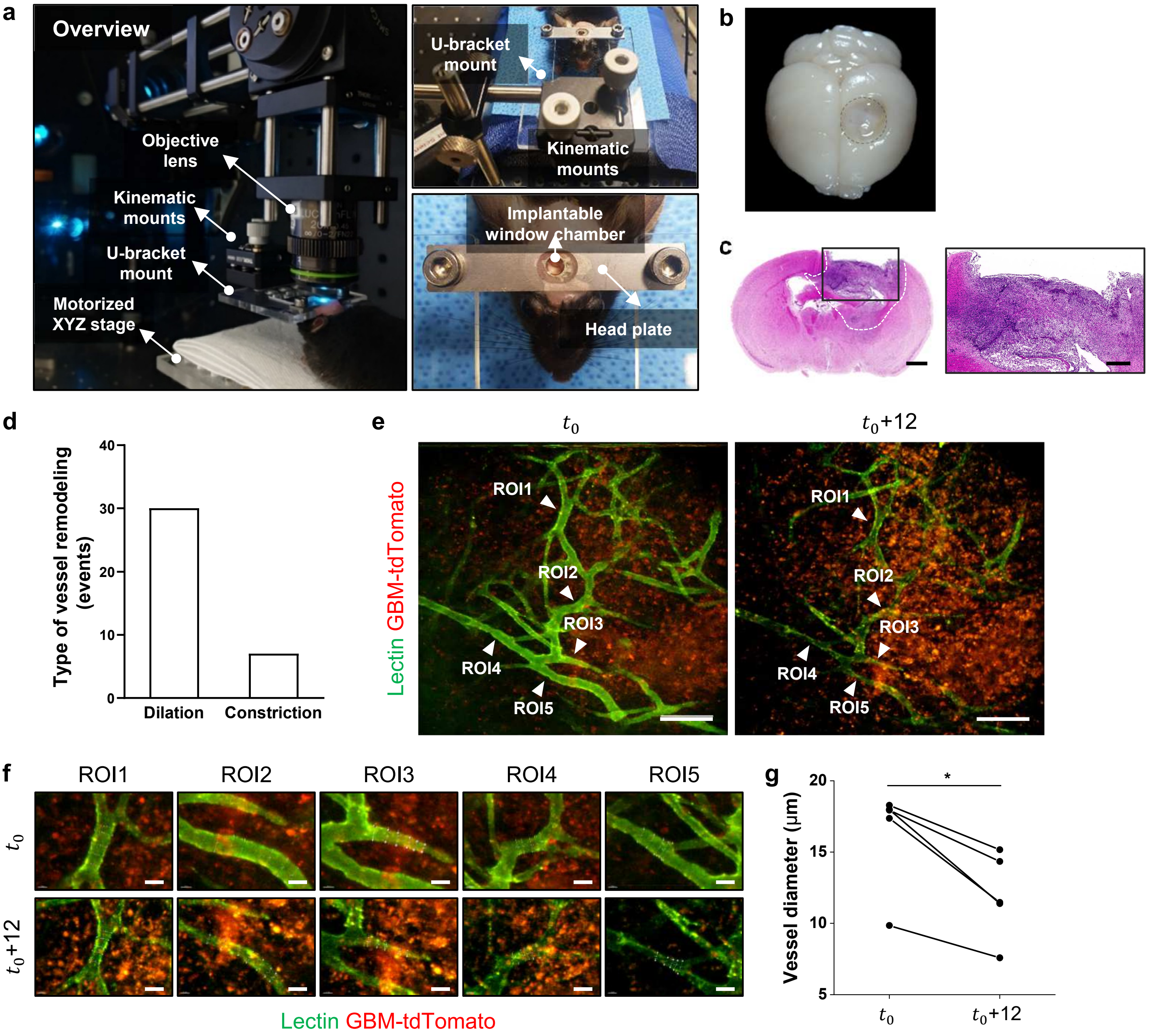
a
Overview
Objective lens
Kinematic mounts
U-bracket mount
Motorized XYZ stage
U-bracket mount
Kinematic mounts
Implantable window chamber
Head plate
b
c
d
Type of vessel remodeling (events)
40
30
20
10
0
Dilation
Constriction
e
t_0
t_0+12
Lectin GBM-tdTomato
ROI1
ROI2
ROI3
ROI4
ROI5
f
ROI1
ROI2
ROI3
ROI4
ROI5
t_0
t_0+12
Lectin GBM-tdTomato
g
Vessel diameter (μm)
20
15
10
5
*
t_0
t_0+12

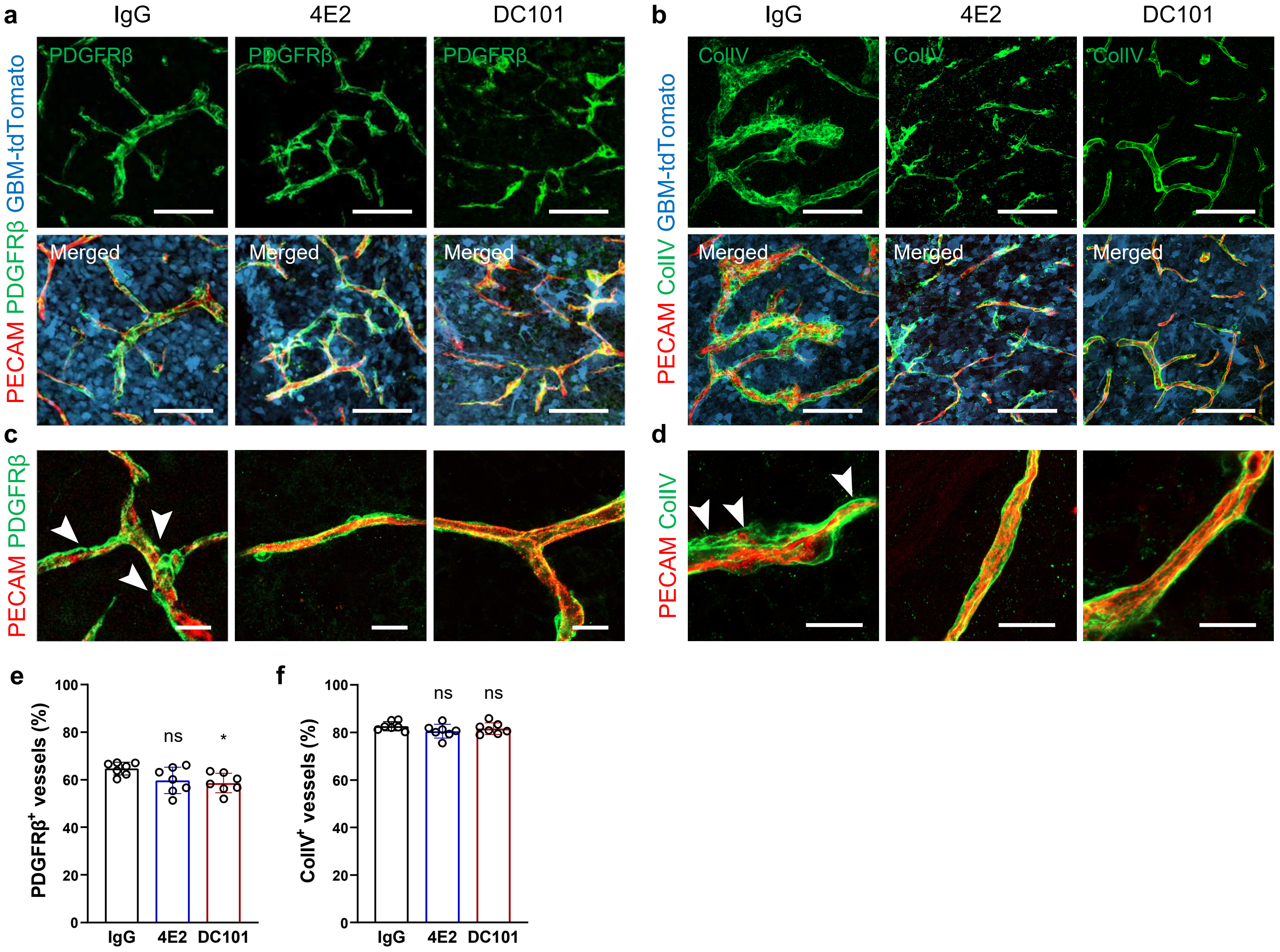
a
IgG
4E2
DC101
PDGFRβ
Merged
PECAM PDGFRβ GBM-tdTomato
b
CollV
Merged
PECAM CollV GBM-tdTomato
c
PECAM PDGFRβ
d
PECAM CollV
e
PDGFRβ+ vessels (%)
ns
*
100
80
60
40
20
0
IgG
4E2
DC101
f
CollV+ vessels (%)
ns
ns

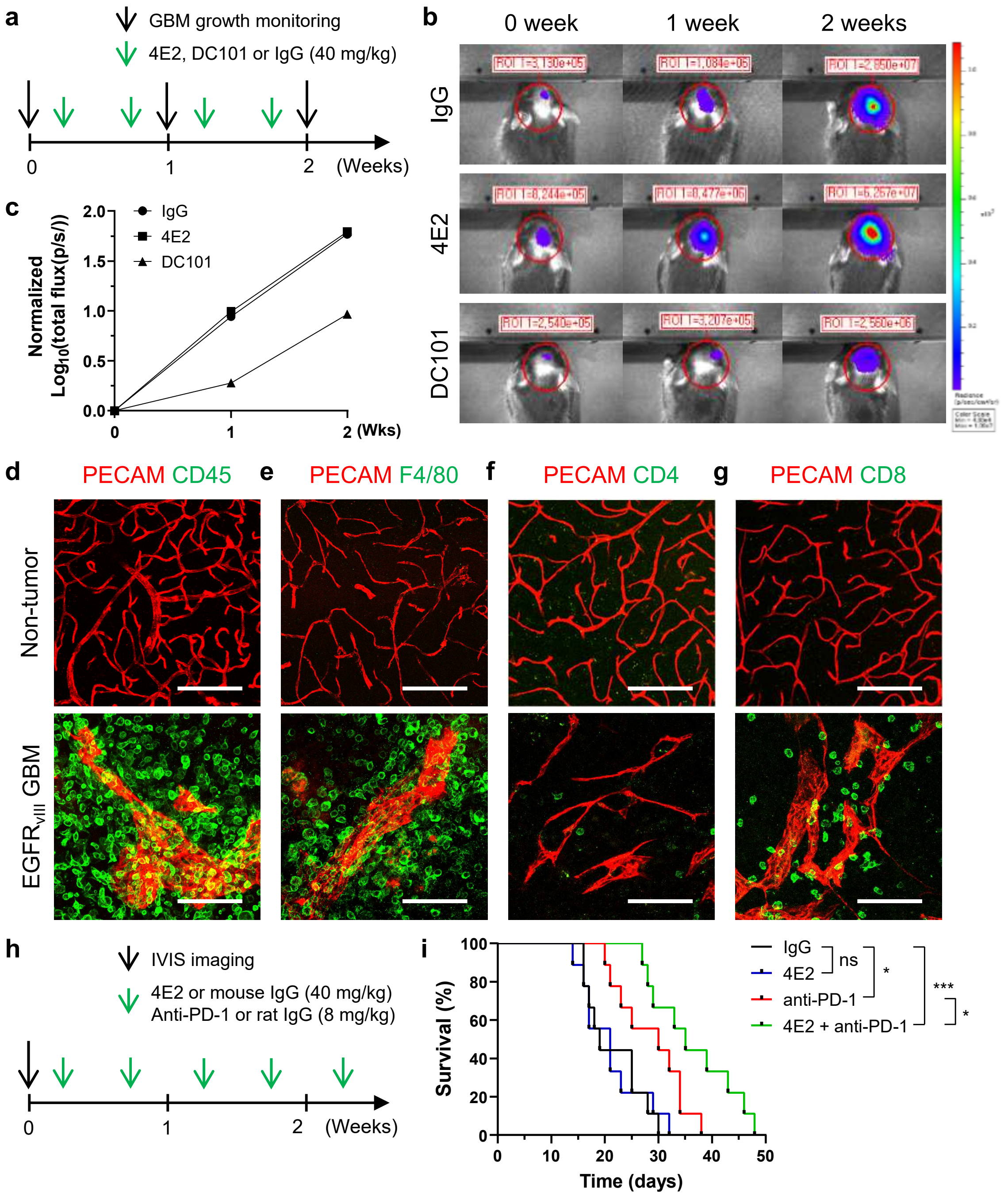
a
GBM growth monitoring
4E2, DC101 or IgG (40 mg/kg)
0
1
2
(Weeks)
b
0 week
1 week
2 weeks
IgG
4E2
DC101
c
Normalized Log10(total flux(p/s/))
IgG
4E2
DC101
2 (Wks)
d
PECAM CD45
e
PECAM F4/80
f
PECAM CD4
g
PECAM CD8
Non-tumor
EGFRvIII GBM
h
IVIS imaging
4E2 or mouse IgG (40 mg/kg)
Anti-PD-1 or rat IgG (8 mg/kg)
(Weeks)
i
Survival (%)
Time (days)
IgG
4E2
anti-PD-1
4E2 + anti-PD-1
ns
*
***

Supplementary Fig. 7

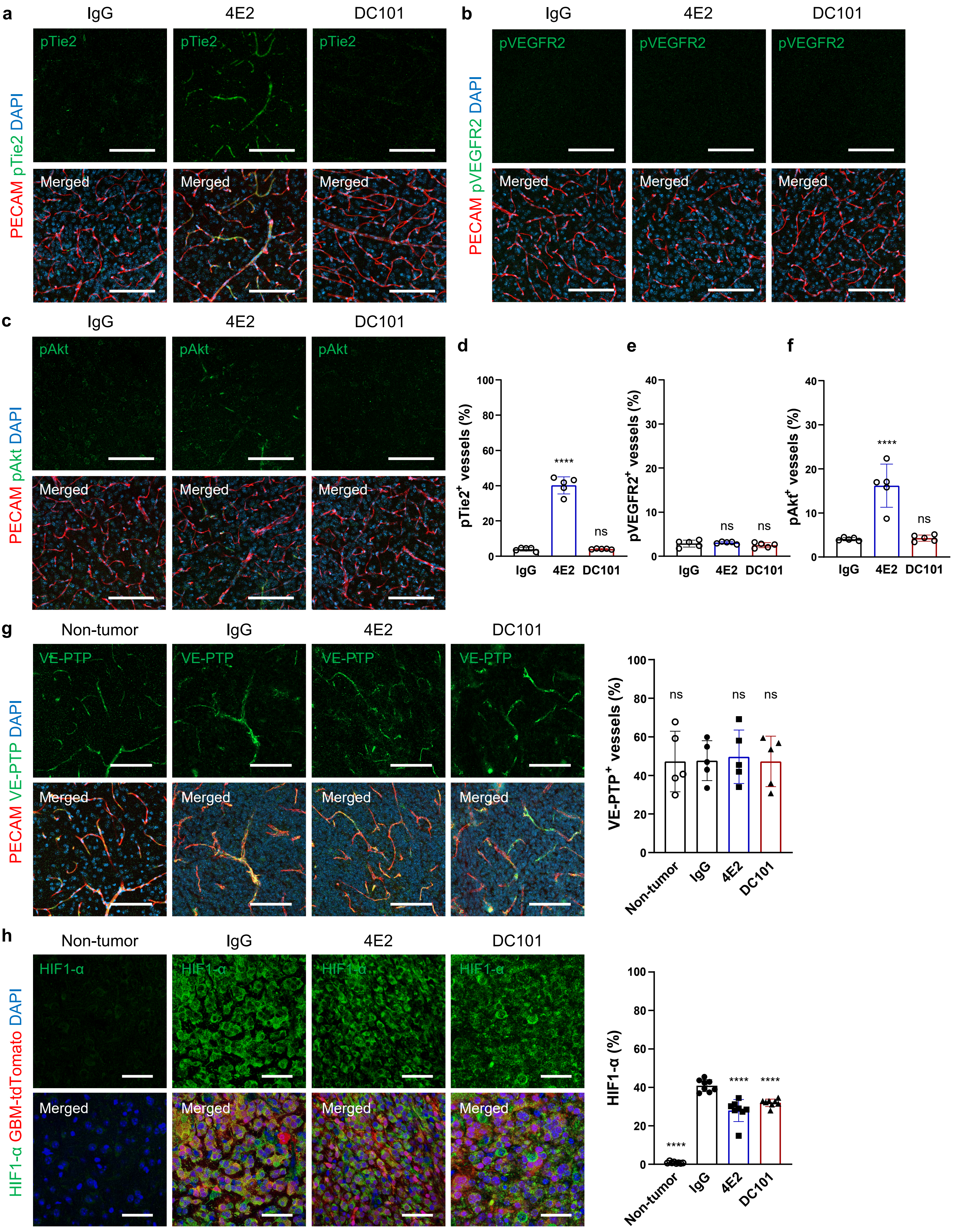

Supplementary Fig. 1. EGFR$_{VIII}$ GBM mouse model represents histopathologic features of grade IV glioma

(**a**) Breeding of mice carrying *tdTomato* and *luciferase* genes for tracking tumor cells *ex vivo* and *in vivo*. (**b**) Survival curves for $EGFR^{VIII/VIII}$ (n = 50) and $EGFR^{WT/WT}$ (n = 30) mice after genetic manipulation. P = 0.0303. (**c**) Macroscopic images showing normal brain of $EGFR^{WT/WT}$ and tumor-bearing brain of $EGFR^{VIII/VIII}$. (**d**) Coronal T2-weighted magnetic resonance images highlighting EGFR$_{VIII}$ GBM with heterogeneous high-signal-intensity mass. (**e-h**) H&E staining images for histopathologic features of grade IV glioma: (**e**) Necrosis with or without pseudo-palisades, (**f**) microvascular proliferation (MVP, arrowheads), (**g**) glomeruloid microvascular proliferation (GMVP, arrowheads), and (**h**) invasion. Note similar histopathological features between of EGFR$_{VIII}$ GBM and GBM from patients. N, necrosis; T, tumor region. (**i-j**) H&E staining images for nuclear atypia (**i**) and mitotic activity (**j**) in GL261 glioma, EGFR$_{VIII}$ GBM and GBM from patients. White arrowheads indicate mitotic chromatin condensation. Scale bars: 1 mm (**c**, **d**), 100 μm (**e-h**), 20 μm (**i-j**).

Supplementary Fig. 2. Increased vascular heterogeneity without sprouting activity in a spontaneous GBM model

(**a**) Immunostaining for PECAM-positive vessels in non-tumor brain, GL261 glioma, and EGFR$_{VIII}$ GBM. (**b**) Immunostaining for PDGFRβ-positive pericytes. (**c**) Immunostaining of collagen type IV (ColIV)-positive basement membrane. (**d-f**) Quantification of vessel area (**d**, n = 10 per group), vessel diameter (**e**, n = 10 per group), and vessel branching point (**f**, n = 8 or 10 per group) in non-tumor brain, GL261 glioma, and EGFR$_{VIII}$ GBM. Quantification data for EGFR$_{VIII}$ GBM here are also used in Fig. 1d-f. Note the biggest deviations in EGFR$_{VIII}$ GBM. (**g-h**) Gross image of Extravasated Evans blue (EB, **g**) and immunostaining of Evans blue at the tumor periphery (**h**). (**i**) Immunostaining of ESM1 in P6 retina, non-tumor brain, and the periphery and center of EGFR$_{VIII}$ GBM. (**j**) Panorama view showing a gradual increase in VEGFR2 levels in vessels from the non-tumor area toward the GBM center. Scale bars: 100 μm (**a-c**, **h**, **i**), 1 mm (**g**), 200 μm (**j**). ns, not significant; *, P < 0.05; **, P < 0.01; ****, P < 0.0001 vs Non-tumor.

Supplementary Fig. 3. Tumor vessels are dilated in human GBM.

Vascular comparison between the periphery and the center of human GBMs. (**a**) H&E staining of brain tissues from control human subjects and paired specimens from patients with GBM.

Peripheral GBM region (upper) and central GBM region (lower). (**b**) Immunostaining for PECAM-positive blood vessels and (**c**) quantification of vessel diameter in normal brain (n = 40), GBM periphery and GBM center (n = 60 per group). (**d**) Comparison of vessel diameter in paired tissues from each patient (n = 10 per group). Scale bars: 100 μm (**a-b**). *, $P < 0.05$; **, $P < 0.01$; ****, $P < 0.0001$.

Supplementary Fig. 4. Dynamics of tumor vessel remodeling in the GBM periphery

(**a**) Photograph of imaging setup and anesthetized GBM mouse implanted with the tube imaging for intravital imaging (**b**) Macroscopic bright field image of mouse brain. (**c**) H&E staining of coronal section of mouse brain bearing $EGFR_{VIII}$ GBM (broken white lines) after the removal of window chamber (left). Magnification of boxed area in (**b**) showing the depth of window chamber inserted below the skull surface (right). (**d**) Quantification of vessel dilation and constriction in GBM. (**e**) Vasoconstriction in control GBM and (**f**) high magnification of region of interests (ROIs) with time interval of 12 hours (t_0 ~ t_0+12). (**g**) Quantification of vessel diameter. Scale bars: 1 mm (**c**, left), 500 μm (**c**, right), 200 μm (**e**), 20 μm (**f**). *, $P < 0.05$.

Supplementary Fig. 5. 4E2 and DC101 suppress tumor vascular abnormalities

(**a-d**) Vascular coverage in $EGFR_{VIII}$ GBM 2 weeks after IgG, 4E2, and DC101 treatment. (**a**) Coverage of PDGFRβ-positive pericytes and (**b**) ColIV-positive basement membrane in the tumor center regions. (**c**) High-magnification images of pericytes and (**d**) basement membrane. Arrowheads indicate their loose contact on GBM vessels. (**e**) Quantification of the coverage of PDGFRβ-positive pericytes (n = 7 per group) and (**f**) ColIV-positive basement membrane (n = 7 per group). Scale bars: 100 μm (**a**, **b**), 20 μm (**c**, **d**). ns, not significant; *, $P < 0.05$ vs IgG.

Supplementary Fig. 6. 4E2 combined with anti-PD-1 antibody extended survival of GBM-bearing mice

(**a**) Experimental schedule of GBM growth monitoring and administration of antibodies. (**b**) *In vivo* bioluminescence imaging and (**c**) quantification of luminescent intensity (n = 6 per group). (**d-g**) Immunostaining for CD45 (**d**), F4/80 (**e**), CD4 (**f**), and CD8 (**g**) in non-tumor brain and $EGFR_{VIII}$ GBM. (**h**) Experimental schedule of antibody administration for survival tests. (**i**) Survival curves of GBM-bearing mice treated with IgG, 4E2, anti-PD-1 antibody, or both 4E2

and anti-PD-1 antibody (n = 9 per group). Scale bars: 100 μm (**d-g**). ns, not significant; *, P < 0.05; ***, P < 0.001.

Supplementary Fig. 7. 4E2 activates Tie2-Akt signaling in non-tumor brain tissues
(**a-c**) Non-tumor brain vessels (contralateral to a $EGFR_{VIII}$ GBM) were imaged in $EGFR_{VIII}$ GBM 2 weeks after IgG, 4E2, and DC101 treatment. (**a**) Increased phospho-Tie2 immunoreactivity in non-tumor brain vessels by 4E2 treatment. (**b**) No detectable phospho-VEGFR2 signal in non-tumor brain vessels by 4E2 treatment. (**c**) Increased phospho-Akt immunoreactivity in non-tumor brain vessels by 4E2 treatment. (**d-f**) Quantification of phospho-Tie2 (**d**, n = 5 per group), phospho-VEGFR2 (**e**, n = 5 per group), and phospho-Akt (**f**, n = 5 per group) in non-tumor brain treated with IgG, 4E2, and DC101. (**g**) Immunostaining and quantification of VE-PTP in non-tumor brain and $EGFR_{VIII}$ GBM treated with IgG, 4E2, and DC101 (n = 5 per group). (**h**) Immunostaining and quantification of HIF1-α in non-tumor brain and $EGFR_{VIII}$ GBM treated with IgG, 4E2, and DC101 (n = 8 per group). Scale bars: 100 μm (**a-c**, **g**), 50 μm (**h**). ns, not significant; ****, P < 0.0001 vs IgG.
